# Profiling of Extracellular Vesicles of Non‐Small Cell Lung Cancer Reveals Proteins Associated With Osimertinib Resistance

**DOI:** 10.1002/jev2.70219

**Published:** 2026-01-25

**Authors:** Albano Cáceres‐Verschae, Petra Hååg, Sofia Joelsson, Per Hydbring, Bo Franzén, Ákos Végvári, Inger Johanne Z. Eide, Nupur Agarwal, Siddharth Sourabh Sahu, Fredrik Stridfeldt, Luigi De Petris, Apurba Dev, Simon Ekman, Odd Terje Brustugun, Rolf Lewensohn, Kristina Viktorsson

**Affiliations:** ^1^ Dept of Oncology‐Pathology Karolinska Institutet Stockholm Sweden; ^2^ Dept of Medical Biochemistry and Biophysics Karolinska Institutet Stockholm Sweden; ^3^ Section of Oncology Vestre Viken Hospital Trust Drammen Norway; ^4^ Dept of Cancer Genetics, Institute for Cancer Research, Norwegian Radium Hospital, Oslo University Hospital, Dept of Clinical Medicine University of Oslo Oslo Norway; ^5^ Dept of Applied Physics, School of Engineering Sciences KTH Royal Institute of Technology Stockholm Sweden; ^6^ Theme Cancer, Patient Area Head, Neck, Lung and Skin Cancer Karolinska University Hospital Stockholm Sweden; ^7^ Division of Solid‐State Electronics, Department of Electrical Engineering Uppsala University Uppsala Sweden

**Keywords:** biomarkers, extracellular vesicles, mutant epidermal growth factor receptor, non‐small cell lung cancer, osimertinib

## Abstract

Precision cancer medicine with small tyrosine kinase inhibitors (TKIs) directed toward oncogenic drivers, are important treatment regimens for solid tumours. The epidermal growth factor receptor (EGFR)‐TKI osimertinib is a preferred therapy for patients with non‐small cell lung cancer (NSCLC) driven by activating mutations in *EGFR*, unfortunately responses are heterogeneous. This calls for non‐invasive methods to predict or monitor treatment response, for example, via biomarker analyses in blood. To reveal such putative biomarkers, we analysed the proteome of extracellular vesicles (EVs) from osimertinib resistant or responsive NSCLC cells in vitro and from EVs isolated from serum samples of NSCLC patients treated with osimertinib in second line within the phase II clinical trial TREM. The protein cargo of the EVs was analysed by mass spectrometry (MS) and proximity extension assay (PEA). Western blotting, ELISA and single vesicle analysis was performed to validate and further confirm the expression of certain proteins. MS profiling of the NSCLC cells and their released EVs revealed a protein signature associated with osimertinib refractoriness. Among them were CSPG4, HSPG2, MCAM, L1CAM, TAGLN, THBS1 and TNC. GO‐pathway analysis related several of these proteins to the focal adhesion and proteoglycan in cancer pathways. Some of these proteins, including CSPG4, which when suppressed by transient siRNA transfection in NSCLC cells resulted in reduced cell viability, were expressed also in EVs from serum of the NSCLC patients. Moreover, PEA profiling of the serum‐isolated EVs revealed signatures associated with immune cells, best response and/or progression‐free survival, including PD‐L1, CD73/NT5E, FR‐alpha/FOLR1, LAMP3, FASLG1 and ANXA1. In summary, we demonstrate that protein profiling of EVs in relation to osimertinib refractoriness has the potential to identify possible biomarkers that can indicate osimertinib treatment resistance, for example, CSPG4, HSPG2, TAGLN, TNC, THBS1, ANXA1 and CD73/NT5E. Studies in expanded cohorts should be conducted to further validate these putative osimertinib biomarkers.

## Graphical abstract

Non‐invasive biomarkers are needed to further personalize EGFR tyrosine kinase inhibitor (EGFR TKI) osimertinib treatment in non‐small cell lung cancer (NSCLC) patients. By analysing the proteome of extracellular vesicles (EVs) isolated from an osimertinib resistant NSCLC cell line and from serum of patients given osimertinib in second line within the TREM trial (NCT02504346), we here revealed proteins putatively associated with response and outcome to osimertinib treatment.

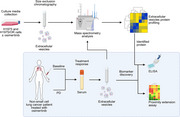

**Abbreviations**
µM: micromolar
Alix: ALG‐2‐interacting protein X
ANXA1: annexin A1
APC: allophycocyanin
BRAF: B‐Raf proto‐oncogene, serine/threonine kinase
CA9: carbonic anhydrase 9
CADM1: cell adhesion molecule 1
CCNY: cyclin Y
CD73/NT5E: cluster of differentiation 73 /5'‐nucleotidase
CD9: cluster of differentiation 9
CEACAM1: carcinoembryonic antigen‐related cell adhesion molecule 1
COL12A1: collagen alpha‐1(XII) chain
CSPG4: chondroitin sulfate proteoglycan 4
ctDNA: circulating tumour DNA
CXCL11: C‐X‐C motif chemokine 11
Del 19: deletion of the exon 19 in the EGFR protein
EGF: epidermal growth factor
EGFR: epidermal growth factor receptor
EGFR‐mut: EGFR mutation
EGFR‐TKI: epidermal growth factor receptor tyrosine kinase inhibitor
ELISA: enzyme‐linked immunosorbent assay
EMT: epithelial‐to‐mesenchymal transition
ESCRT: endosomal sorting complexes required for transport
EVs: extracellular vesicles
FAK: focal adhesion kinase
FASLG: fas ligand
FDR: false discovery rate
FGFR: fibroblast growth factor receptor
FR‐alpha/FOLR1: folate receptor 1
FSTL1: follistatin‐related protein 1
GAPDH: glyceraldehyde‐3‐phosphate dehydrogenase
GO: gene ontology
HER2: human epidermal growth factor receptor 2
HGFR: hepatocyte growth factor receptor
HMNC1: hemicentin 1
HSPG2: heparan sulfate proteoglycan 2
IC50: inhibiting concentration by 50%
ISEV: International Society for Extracellular Vesicles
KNN: K‐nearest neighbour
KRAS: KRAS proto‐oncogene
L1CAM: neural cell adhesion molecule L1
LAMP3: lysosome‐associated membrane glycoprotein 3
LC: lung cancer
LC‐MS/MS: liquid chromatography‐tandem mass spectrometry
LOXL2: lysyl oxidase homolog 2
LUAD: lung adenomcarcinomas
MATN2: matrilin 2
MCAM: cell surface glycoprotein MUC18
MET: hepatocyte growth factor receptor
Mo.: months
MS: mass‐spectrometry
nm: nanometres
NPX: normalized protein eXPression
NSCLC: non‐small cell lung cancer
NTA: nanoparticle tracking analysis
OR: osimertinib resistance
OS: overall survival
Osi: osimertinib
PCM: precision cancer medicine
PCR: polymerase chain reaction
PD: progressive disease
PD‐L1: programmed death‐ligand 1
PEA: proximity extension assay
PFS: progression‐free survival
pg/mL: picograms per millilitre
PIK3CA: phosphatidylinositol‐4,5‐bisphosphate 3‐kinase catalytic subunit alpha
PR: partial response
PTPRG: receptor‐type tyrosine‐protein phosphatase gamma
PVDF: polyvinylidene difluoride
qPCR: quantitative polymerase chain reaction
RAB32: ras‐related protein Rab‐32
RTK: receptor tyrosine kinase
SCAMP3: secretory carrier‐associated membrane protein 3
SCLC: small cell lung cancer
SD: stable disease
SEC: size exclusion chromatography
SRC: tyrosine‐protein kinase Src
SVM: support vector machine
TAGLN: transgelin
TEM: transmission electron microscopy
THBS1: thrombospondin 1
TKI: tyrosine kinase inhibitor
TLR3: toll‐like receptor 3
TME: tumour microenvironment
TMT: tandem mass tag
TNC: Tenascin
TREM: multi‐institutional phase 2 single‐arm study TREM (NCT02504346)
VWA1: von Willebrand factor A domain‐containing protein 1
XGBoost: extreme gradient boosting
XPNPEP2: X‐prolyl aminopeptidase 2
YAP: yes‐associated protein

## 1 Introduction

Lung cancer (LC) patients with advanced or metastatic disease have an unfortunate outcome and less than 20% of all patients survive 5 years post diagnosis (Bray et al. 2024). About 85% of all LC cases are non‐small cell lung cancer (NSCLC) and out of these approximately 40% are lung adenocarcinomas (LUADs). Genomic profiling of LUAD tumours have identified oncogenic drivers, offering targets for precision cancer medicine (PCM) treatments (Passaro et al. 2021; Wang et al. 2021; Meyer et al. 2024). One example is the epidermal growth factor receptor (EGFR), which is found to be mutated in about 10%–50% of all LUAD patients depending on their Caucasian or East‐Asian origin (Midha et al. 2015). The alterations in *EGFR*, for example, exon 19 deletion (amino acids 747–750, the LREA motif) or the point mutation in exon 21 (L858R), which are located in the receptor kinase domain results in a constitutively activation of the receptor (Passaro et al. 2021). Most patients with mutant *EGFR*‐driven tumours initially have a good response to earlier generations of EGFR‐tyrosine kinase inhibitors (EGFR‐TKIs), for example, erlotinib, gefitinib, afatinib and dacomitinib (Passaro et al. 2021; Meyer et al. 2024; Gomez‐Randulfe et al. 2025). However, a large fraction of these patients acquire resistance to the given EGFR‐TKI over time which can be ‘on target’, that is related to alteration of the *EGFR* itself, or be due to ‘off‐target’‐signalling, which is driven by other receptor tyrosine kinases (RTKs) which compensate for a TKI‐blocked EGFR (Passaro et al. 2021; Gomez‐Randulfe et al. 2025). A large proportion of the EGFR‐TKI resistance mechanisms is a result of an *EGFR T790M* point mutation in exon 20. This mutation changes the kinase pocket and prevents first‐generation EGFR‐TKIs from acting (Passaro et al. 2021; Gomez‐Randulfe et al. 2025). The ‘off‐target’ resistance mechanisms on the other hand involve activation of other RTKs as a result of, for example, mutations or amplifications (Nukaga et al. 2017). Moreover, RNA or epigenetic deregulation as well as transformation into small cell lung cancer (SCLC) are other causes of resistance to first‐generation EGFR‐TKIs (Passaro et al. 2021; Kashima et al. 2021).

The third generation EGFR‐TKI osimertinib can exert an effect on the EGFR kinase activity although the compensatory mutation *EGFR T790M* is present (Finlay et al. 2014; Cross et al. 2014; Ballard et al. 2016). Accordingly, osimertinib has been shown to be effective in second‐line when resistance has already arisen toward earlier generation EGFR‐TKIs (Jänne et al. 2015; Mok et al. 2017; Papadimitrakopoulou et al. 2020). Osimertinib is now also given in first line for advanced cases with improved patient outcome, (Soria et al. 2018; Planchard et al. 2019; Ramalingam et al. 2020) and has demonstrated improved survival in patients with resected stage IB to IIIA disease (Tsuboi et al. 2023). Unfortunately, new alterations in the *EGFR* gene can arise resulting in osimertinib refractoriness, and these alterations may differ whether osimertinib is given in first‐ or second‐line (Passaro et al. 2021; Gomez‐Randulfe et al. 2025).

Osimertinib resistance may also, in a similar manner as refractoriness to earlier generation EGFR‐TKIs, be a consequence of activation of bypass signalling via multiple RTKs or their downstream components (Passaro et al. 2021; Gomez‐Randulfe et al. 2025). These signalling pathways include, for example, tyrosine kinase Met/hepatocyte growth factor receptor (c‐MET/HGFR), human epidermal growth factor receptor 2 (HER2), phosphatidylinositol‐4,5‐bisphosphate 3‐kinase catalytic subunit alpha (PIK3CA), B‐Raf proto‐oncogene, serine/threonine kinase (BRAF), KRAS proto‐oncogene (KRAS) and fibroblast growth factor receptor (FGFR) (reviewed in (Passaro et al. 2021; Gomez‐Randulfe et al. 2025)). Resistance to osimertinib can also be caused by alterations in the epithelial‐to‐mesenchymal transition (EMT) signalling via the Yes‐associated protein (YAP) signalling network as shown by us and others (McGowan et al. 2017; Shi et al. 2025). Moreover, we and others earlier reported that transcriptomic alterations associated with osimertinib resistance are, among others, thrombospondin 1 (THBS1) and the Ras‐related protein Rab‐32 (RAB32), with a clear role also for the focal adhesion kinase (FAK) signalling pathway (Kosibaty et al. 2022; Ichihara et al. 2017).

As osimertinib elicits a heterogeneous response in tumour lesions, and since repeated tissue biopsies are challenging in metastatic LUAD cases, alternative methods to accurately evaluate osimertinib response or refractoriness are necessary. Circulating tumour DNA (ctDNA) has thus been used to predict early treatment benefit of osimertinib (Chmielecki et al. 2023; Gray et al. 2023; Rotow et al. 2024; Ruglioni et al. 2025). However, as alterations on RNA or protein level also contribute to osimertinib resistance, ctDNA analysis will not capture and reveal all relevant resistance mechanisms. Here, extracellular vesicles (EVs) isolated from blood could potentially serve as a source of biomarkers as these vesicles contain lipids, RNA and protein cargo that partly reflect their tumour cell of origin (Kalluri and McAndrews 2023; Asao et al. 2023). Moreover, EVs may also alter signalling in the tumour or act as communicators between the tumour and its tumour microenvironment (TME), further strengthening their potential role as carriers of biomarkers (Kalluri and McAndrews 2023; Asao et al. 2023; Lucotti et al. 2022). EVs have also been demonstrated by us and others to hold biomarker potential in the context of mutant EGFR‐driven NSCLC, related to both RNA and protein cargo (Krug et al. 2018; Purcell et al. 2021; Stiller et al. 2021; Sahu et al. 2023; Stridfeldt et al. 2023; Salih et al. 2025; Banijamali et al. 2022; Shintani et al. 2024; Kaźmierczak et al. 2022). Moreover, our research group reported on RNA alterations in EVs isolated from plasma of NSCLC patients (Alexeyenko et al. 2022) when treated with osimertinib after progressing on first line EGFR‐TKI within the phase II clinical trial TREM (Eide et al. 2020). Here, our EV analyses indicated the involvement of EGFR, PI3K, syndecan and glypican signalling networks (Alexeyenko et al. 2022).

In this work, we aimed to identify and characterize EVs protein cargo associated with osimertinib activity or refractoriness, by using mass spectrometry (MS) and affinity‐based proximity extension assay (PEA). We analysed the protein landscape of EVs and cells of an *in vitro* osimertinib responsive and a resistant NSCLC cell line pair expressing the *EGFR T790M* mutation, and from EVs isolated from serum of NSCLC patients within the phase II clinical trial TREM (Eide et al. 2020). All the patients had activating mutations in *EGFR* and had been treated with, and relapsed on, first‐generation EGFR‐TKI with a large proportion of the patients also expressing the *EGFR T790M* resistance mutation. The analysed EVs were from serum samples that had been taken at the start of osimertinib treatment (baseline), and upon progressive disease (PD). Our focus was to find proteins associated with osimertinib resistance, immune signalling, progression‐free survival (PFS) and best response of the patients.

## 2 Material and Methods

### 2.1 Cell Lines and Culture Conditions

In this study, H1975 (CRL‐5908, ATCC, LGC Standards, Middlesex, Great Britain; RRID: CVCL_1511), a NSCLC cell line with *EGFR L858R* and *T790M* mutations (Pao et al. 1996) resistant to first generation EGFR‐TKI (Sordella et al. 2004; Pao et al. 2005) but sensitive to osimertinib (Cross et al. 2014) and a subline, H1975/OR, (developed by the co‐author Prof. O. T. Brustuguns team (McGowan et al. 2017)), were used. For some supplementary experiments, the NSCLC cell line HCC827 (CRL‐2868, ATCC; LGC Standards, Middlesex, Great Britain; RRID:CVCL_2063) (Amann et al. 2005; Mukohara et al. 2005) or the subline HCC827/OR, made in vitro resistant to osimertinib (McGowan et al. 2017) and further characterized by co‐authors Prof. S. Ekman and Assoc. Prof. P. Hydbring team (McGowan et al. 2017; Kosibaty et al. 2022) were used. The parental cell line, HCC827 is reported to have an activating *EGFR* alteration due to an exon 19 deletion (E746_A750del) and to be sensitive to first generation EGFR‐TKIs (Amann et al. 2005; Mukohara et al. 2005; Naumov et al. 2009). All cells were grown in RPMI‐1640 media (GIBCO, Thermo Fisher Scientific, Waltham, MA, USA, cat. #11530586) supplemented with 10% foetal bovine serum (GIBCO, Thermo Fisher Scientific, cat. #10500064) and 2 mM l‐glutamine (GIBCO, Thermo Fisher Scientific, cat. #25030081) (hereafter called complete media). The experiments were conducted on cells which were mycoplasma negative at the time of cryopreservation.

### 2.2 Patient Material

The serum samples used for isolating the EVs were obtained from a cohort of NSCLC patients treated with osimertinib in second‐line at Karolinska University Hospital, Solna, Sweden, within the phase II clinical trial TREM (NCT02504346) (Eide et al. 2020). In total, 21 patients were included in the TREM trial at the Karolinska University Hospital site and their samples were used if available, resulting in 17 patients providing samples at baseline as well as progression and with four only giving samples at progression. Blood samples from the patients were collected in 8 mL yellow capped serum separating tubes with gel and anti‐coagulant factor, respectively. The tubes were inverted gently and incubated upright for 1 h at room temperature to allow coagulation. Following this, the tubes were centrifuged at 2500 g for 10 min at room temperature and the serum was divided into 1.2 mL vials and frozen at −80°C until use. Two vials from each sample taken at baseline or at progression were used. A summary of the patient characteristics is given in Table 1. The Swedish Medical Products Agency (EudraCT; nr; 2015‐000307‐10) and the Regional Ethical Review Board in Stockholm (EPN No. 2016/710‐31/1) approved the study with all patients signing informed consent. Responses were determined by Response Evaluation Criteria In Solid Tumours version 1.1 (RECIST 1.1) criteria (Eide et al. 2020) and defined as progressive disease (PD), stable disease (SD) and partial response (PR). None of the patients in this study reached complete remission (CR).

**TABLE 1:** Molecular and clinical parameters of the cohort.

General characteristics	Overall *n* = 21	EGFR *T790M*‐positive* *n* = 16	EGFR *T790M*‐negative* *n* = 5
Median age [range]—Years	66 [39–81]	69.5 [44–81]	52 [39–71]
Sex			
Female Male	12 (57 %) 9 (43 %)	11 (69 %) 5 (31 %)	1 (20 %) 4 (80 %)
EGFR‐mut. at inclusion			
L585R Del 19 G719X G719X + S768I G719X + L861Q	7 (33 %) 10 (47 %) 1 (5 %) 2 (10 %) 1 (5 %)	5 (32 %) 9 (56 %) 0 (0 %) 1 (1 %) 1 (1 %)	2 (40 %) 1 (20 %) 1 (20 %) 1 (20 %) 0 (0 %)
Previous EGFR‐TKI therapy			
Erlotinib Afatinib Erlotinib + Afatinib	18 (85%) 2 (10 %) 1 (5 %)	14 (88 %) 2 (12 %) 0 (0 %)	4 (80%) 0 (0 %) 1 (20 %)
No. of systemic therapies before Osimertinib			
1 2 ≥3	8 (38 %) 11 (52 %) 2 (10 %)	7 (44 %) 8 (50 %) 1 (6 %)	1 (20 %) 3 (60 %) 1 (20 %)
Progression‐free survival (PFS, Mo.)	9.1 [1.6–25.2]	9.1 [3.7–21.5]	7.4 [1.6–25.2]
Overall survival (Os, Mo.)	22.9 [7.5–51.1]	22.3 [8.5–51.1]	28.9 [7.5–45.1]
Best response			
Partial response (PR) Stable disease (SD) Progressive disease (PD)	12 (57 %) 8 (38 %) 1 (5 %)	10 (63 %) 6 (38 %) 0 (0 %)	2 (40 %) 2 (40 %) 1 (20 %)

*Notes*: All patients from which serum was used for isolation of EVs and subsequent protein profiling had stage IV adenocarcinoma with different activating *EGFR* mutation status at study inclusion with or without *EGFR T790M*.

Abbreviations: EGFR mut., epidermal growth factor receptor mutation; EGFR‐TKI, EGFR tyrosine kinase inhibitor; Mo., months; OS, overall survival; PD, progressive disease; PFS, progression‐free survival; PR, partial response; SD, stable disease, X, any amino acid.

Although all of the TREM study patients had activating mutations in *EGFR* and had been given EGFR‐TKI regimen(s) prior to osimertinib (Table 1), the subset of the patients included at Karolinska University Hospital in this trial and from which samples were used in this work, had more patients with an *EGFR T790M* positive tumour compared to the entire TREM study cohort (76% vs. 60%). Patients with an *EGFR T790M* negative tumour were fewer in both cohorts (24% vs. 26%) which was expected since osimertinib was given in second line. The median progression‐free survival (PFS; 9.1 months vs. 8.9 months) were rather similar between the Karolinska University Hospital patients and the entire TREM cohort cohorts despite the difference in the cohort sizes. In contrast, overall survival (OS) differed slightly with the patients treated at Karolinska University Hospital having median OS of 22.9 months versus 17.9 months in the entire TREM study population. With respect to best response, both cohorts were similar regarding the fraction of patients with SD while the Karolinska University Hospital cohort had more patients with PR, and only one patient with PD (Table 1 and Eide et al. [Eide et al. 2020], respectively).

### 2.3 Isolation of Extracellular Vesicles From Cell Culture Media

Cell culture media of H1975 or H1975/OR cells (from two T175 flasks with 2.6 × 10^6^ seeded cells per flask per condition) were used for isolation of extracellular vesicles (EVs). At 48 h post seeding, 20 mL fresh RPMI media supplemented with 10% exosome‐free FBS (GIBCO, Thermo Fisher Scientific, cat. #A2720801) were added to each flask. After another 48 h incubation with or without 0.1 µM osimertinib, the cell culture media was collected and centrifuged at 300 g for 5 min, followed by a centrifugation at 2000 g for 10 min to remove cell debris and large particles, as earlier described (Kowal et al. 2016). Subsequently, the samples were concentrated using Amicon ultra 15 mL centrifugal filters (100 kDa cutoff, Merck Millipore, Burlington, MA, USA) to approximately 500 µL/sample. To isolate the EVs, the samples were thereafter loaded on qEV original gen2, 70 nm (Izon Science, Lyon, France) size exclusion chromatography (SEC) columns. Fractions 1–5 (500 µL each) following a void volume of 2.8 mL were gathered using an automatic fraction collector (AFC2; IZON), pooled and concentrated in Amicon ultra 0.5 mL centrifugal filters (100 kDa cutoff, Merck Millipore) to ∼150 µL/sample. Particle size and concentration were assessed by nanoparticle tracking analysis (NTA) on a NS300 instrument (Malvern Panalytical, Malvern, UK), using five videos of 30 s with the camera level 11 and with detection threshold set to 3 in which about 35–120 particles/frame were analysed. For isolating EVs from HCC827/OR cell culture media, a similar procedure was followed. H1975 or H1975/OR cell viability was determined by trypan blue exclusion, and the amount of EVs after 48 h in untreated cell cultures or after osimertinib treatment for 48 h, was calculated using the NTA particles/mL value and the total volume of the cell culture media at the time of harvest.

### 2.4 Isolation of Extracellular Vesicles From Serum

The serum samples from the patients were thawed, centrifuged at 720 g (10 min on Mikro 200R, Hettich, Stockholm, Sweden) and filtered using 0.22 µm, 13 mm diameter Acrodisc syringe filters (Pall Corporation, VWR, Spånga Sweden). The individual starting volumes of the serum ranged from 350 µL to 1.1 mL. After filtering, the sample volumes were adjusted to 500 µL which subsequently was used for the EV isolation on the qEVoriginal 70 nm (legacy) columns (Izon Science). After the void volume of 2.8 mL, the following five fractions of 500 µL were collected using AFC (see above), pooled and concentrated to ∼200 µL/sample by Amicon Ultra‐4 (3 kDa cutoff, Merck Millipore). NTA assessment was done to reveal particle sizes (in nm) and particles/mL diluting the samples 1:100 in PBS. The NTA measured three videos of 60 s with camera level 10–16, detection threshold set to 5 and 38–280 particles/frame.

### 2.5 Western Blot Assessment of Extracellular Vesicles and Cell Extracts

Western blot was carried out on H1975, H1975/OR and HCC827/OR cells, their released EVs or from a subset of the serum‐isolated EVs of the TREM‐study patients. The samples were lysed in RIPA buffer (50 mM Tris‐HCl, pH 7.4, 150 mM NaCl, 1% Triton X‐100, 5 mM EDTA pH 8, 0.1% SDS (Merck Millipore, cat. #20‐188) to which PhosSTOP and protease inhibitors (Roche, Stockholm, Sweden, cat. #4906845001 and cat. #11836170001, respectively) were added. Proteins were separated on 4%–12% NuPAGE gels (Invitrogen, MA, USA, cat. #NP0321) and transferred to nitrocellulose (Li‐COR, Bad Homburg, Germany, cat. #926‐31092) or polyvinylidene difluoride (PVDF) membranes (GE Healthcare, Freiburg, Germany, cat. #RPN303F). For blocking the membranes and diluting the antibodies, odyssey blocking solution (Li‐COR, cat. #927‐60001) in TBS‐tween 0.01% 1:1 or skim milk 5% in TBS‐tween 0.01% was used. The primary antibodies applied were: CD9 (Cell Signalling Technologies, Danvers, MA, USA, cat. #13403, RRID: AB_2732848; 1:500‐1:1000); alix (Abcam, Cambridge, United Kingdom, cat. #ab186429, RRID: AB_2754981; 1:1000); syntenin‐1 (Abcam, cat. #ab133267, RRID: AB_11160262; 1:1000), calnexin (Cell Signalling Technologies, cat. #2433, RRID: AB_2243887; 1:1000, flotillin‐1 (Cell Signalling Technology, cat. #18634, RRID:AB_2773040, 1:1000). Secondary antibodies were anti‐Rabbit 680 IRDye (cat. #926‐68071, RRID: AB_10956166; 1:15,000), anti‐mouse 800 IRDye (cat. #926‐32210, RRID: AB_621842; 1:15,000) both from Li‐COR or anti‐rabbit (cat. #7074, RRID: AB_2099233; 1:10,000) or anti‐mouse (cat. #7076, RRID: AB_330924; 1:10,000) HRP conjugated antibodies from Cell Signalling Technologies. The western blot images were scanned with the Li‐COR Clx or Odyssey Sa (Li‐COR) equipment or iBright FL1500 (Thermo‐Fisher) and the images were further processed with Fiji (ImageJ, RRID: SCR_003070) software (Schindelin et al. 2012) or Image studio ver. 3.1 (Li‐COR). The images were adjusted with Adobe Photoshop (2023; Acrobat Reader).

### 2.6 Transmission Electron Microscopy Analyses of Extracellular Vesicles

EVs size and morphology was assessed by transmission electron microscopy (TEM) at the Electron Microscopy core facility (Department of Laboratory Medicine, Karolinska Institutet). For the analysis, the samples were applied on copper grids (Ted Pella, cat. 01814‐F) and processed essentially as earlier described (Mkrtchian et al. 2021). Thus, after incubating for 30 s, the grid with attached material was rinsed in MilliQ water followed by addition of 2% uranyl acetate for negative staining. A Hitachi HT7700 transmission electron microscope (Hitachi High‐Technologies Corporation, Tokyo, Japan) with ×40,000 or ×80,000 magnifications was used for imaging with capturing on a Veleta CCD camera (Olympus Soft Imaging System). The images were cropped and processed using Adobe Photoshop (2023; Acrobat Reader).

### 2.7 Mass Spectrometry Profiling of Extracellular Vesicle Protein Cargo

EVs isolated from three biological replicates of H1975 or H1975/OR cells were analysed by liquid chromatography‐tandem mass spectrometry (LC‐MS/MS). The procedure is given with full details in Supporting Information. Briefly, EVs were solubilized in a buffer with 8M urea (Sigma–Aldrich, cat. #51457) to which protease and phosphatase inhibitors (Pierce, cat. #78440) were added. The samples were sonicated and bicinchoninic acid assay (BCA) assay (Thermo Fisher Scientific, cat. #23225) was used to estimate the protein concentration. For the subsequent analysis, 10 µg of total protein was reduced, alkylated, and digested using endoproteinase Lys‐C (Wako, Japan, cat. #125‐05061) and trypsin (Promega, cat. #V5113). To stop the digestion, formic acid (Sigma–Aldrich, cat. #533002) was added. After desalting the samples on a C18 HyperSep plate (Thermo Fisher Scientific, cat. #60300‐425), the samples were dried. Solubilized peptides were injected onto a 50 cm long EASY‐Spray C18 column for reversed‐phase LC separation using an Ultimate 3000 nanoUPLC system coupled to a Q Exactive HF hybrid quadrupole‐Orbitrap mass spectrometer (Thermo Fisher Scientific). The raw MS data were searched against a human consensus protein database (SwissProt, v.2023‐02‐09) using the Amanda 2.0 search engine incorporated in the Proteome Discoverer v3.0 software (Thermo Fisher Scientific). Proteins were taken for further analyses if identified by at least two peptide sequences. The protein quantification was based on the precursor peptide intensities and the expression level of proteins in the samples studied were compared by their raw abundance value. EV‐samples isolated from serum of two NSCLC patients (Ptn. #3 and Ptn. #5) included in the cohort (Table 1; Figure S5A) were also analysed by LC‐MS/MS using the same steps as above, in which 1.2 × 10^9^ EVs per sample were lysed and 2.5 µg protein was studied. The proteins identified in the serum‐isolated EVs were related to proteins identified in the EVs from the osimertinib resistant cell line H1975/OR. They were also compared to the Vesiclepedia (www.microvesicles.org) to confirm EVs origin (Figure S7).

### 2.8 Mass Spectrometry Protein Profiling of Cell Extracts

H1975 and H1975/OR cell extracts obtained from untreated cells or cells subjected to 0.1 µM osimertinib for 48 h were also studied by LC‐MS/MS. The full details of the analyses are found in the **Supporting Information**. Briefly, a 7.5 M urea buffer with addition of protease and phosphatase inhibitors were used to dissolve the cell pellets. An aliquot of 25 µg/sample was trypsin digested into peptides which was preceded by reduction and alkylation. The samples were cleaned on a HyperSepC18 plate (Thermo Fisher Scientific) and dried using a Vacufuge vacuum concentrator (Eppendorf). For the tandem mass tag (TMT) labelling, the solubilized samples in 70 µL 50 mM triethylammonium bicarbonate (TEAB) buffer and 100 µg of TMTpro reagents (Thermo Fisher Scientific) in 30 µL anhydrous acetonitrile were mixed and incubated for 2 h at rotation after which the reaction was stopped by addition of hydroxylamine (0.5% final concentration). One analytical sample was prepared from all the biological replicates to be analysed, which subsequently was dried and cleaned on a C18 StageTip cartridge. High‐pH reversed‐phase liquid chromatography was applied to fractionate the TMTpro‐labelled peptides and ∼2 µg of the concatenated eight fractions from the 12 analytical samples combined, which represented the peak peptide elution, was injected onto a 50 cm EASY‐Spray C18 column. The LC‐MS/MS data acquisition by an Q Exactive HF hybrid quadrupole‐Orbitrap mass spectrometer was similar as described above, except that a higher collisional energy was applied to promote dissociation of the TMTpro reporter ions of the top 17 precursor ions. Search parameters for the protein identification and quantification in the Proteome Discoverer v3.0 (Thermo Fisher Scientific) was done as described in **Supporting Information**, in which TMTpro on lysine and N‐termini, deamidation of asparagine and glutamine, and methionine oxidation was set as dynamic modifications, and carbamidomethylation of cysteine was put as fixed. For identification of proteins at least two peptides were used, and the TMTpro reporter ion abundances of the peptides were used for quantification of the corresponding proteins. The results were initially filtered for 5% FDR by the Percolator node in Proteome Discoverer.

### 2.9 Proximity Extension Assay of the Proteome of Extracellular Vesicles

The protein cargo of serum isolated EVs were analysed by proximity extension assay (PEA) (Olink Proteomics AB, Uppsala, Sweden) using the Oncology II and Immuno‐Oncology panels. Four of the patients (Ptn. #6, #11, #12 and #13) did not have available baseline samples and hence were excluded in the best response and PFS associated EV protein analyses. The EVs (∼1.9 × 10^7^ particles /sample) were lysed in a 5× RIPA buffer to a final concentration of 1× RIPA. A sample with only RIPA buffer without EVs served as negative control for the assay. The PEA assessment was done at the SciLifeLab Affinity Proteomics facility at Uppsala University, Uppsala, Sweden. Initial data processing and interplate‐control normalization was made by the Olink Wizard for GENEX software (MultiD Analyses AB, Gothenburg, Sweden), generating an arbitrary relative quantification unit called Normalized Protein eXpression (NPX) values, which were used in the next analysis steps (for details see: 1096‐olink‐data‐normalization‐white‐paper.pdf). Out of the total 184 proteins in the two PEA panels, 18 proteins were overlapping resulting in 166 unique proteins being evaluated. To be included in the further analysis, the protein had to be expressed above the RIPA buffer negative control in 60% or more of the EV samples in the cohort. Given these criteria, 118 proteins were analysed.

### 2.10 Enzyme‐Linked Immunosorbent Assay of Chondroitin Sulfate Proteoglycan 4 and Cluster of Differentiation CD73/5’‐Nucleotidase Expressions in Extracellular Vesicle Samples

Expression of chondroitin sulfate proteoglycan 4 (CSPG4) was validated on intact EVs using a human CSPG4 SimpleStep ELISA Kit (Abcam, cat. #ab267807), per manufacturer's manual. A total of 1 × 10^9^ EVs isolated from H1975 or H1975/OR cell culture media (four biological replicates for H1975 and six biological replicates for H1975/OR), HCC827/OR cell culture media (in two biological replicates) or from serum of the NSCLC patients (Table 1) at baseline and progression if available, were analysed as single replicates. The ELISA plate was read at 450 nm (SpectraMax I3, Molecular Devices). The concentration of CSPG4 on the EVs (in pg/mL) was measured using a standard curve as per kit instructions. For the cluster of differentiation 73 (CD73/NT5E) assessment, a subset of the serum EV samples from the NSCLC patients taken at baseline were used. The RIPA‐lysed EV samples (2 × 10^7^ to 2 × 10^8^ particles/sample) were added to an ELISA plate coated with anti‐CD73/NT5E antibody (Thermo Fisher Scientific, cat. #EH5RB) and the reaction carried out as per the manufacturer's instructions, using a standard curve provided by the kit. The levels of CD73/NT5E (pg/mL) in each sample were normalized to ∼1.0 × 10^7^ EVs/sample.

### 2.11 Fluorescent‐Based Single Vesicle Analyses

Programmed Death‐Ligand 1 (PD‐L1), CD73/NT5E or CD9 expression on EVs isolated from the indicated NSCLC patient serum samples at baseline were examined by a fluorescence‐based single‐EV method as described (Sahu et al. 2023; Stridfeldt et al. 2023). Briefly, coverslips were incubated with 1 µg/µL silane‐PEG‐Biotin (Laysian Bio, Inc., AL, USA, cat. #145‐40), blocked with casein and conjugated with biotinylated CD9 (Novus Biologicals, Abingdon, United Kingdom, cat. #NB500‐327B) using a streptavidin linker. A total of 3 × 10^8^ EV particles in 100 µL PBS were incubated followed by addition of the following antibodies (4 nM each): PE‐conjugated anti‐PD‐L1 (Bio‐Techne, Abingdon, United Kingdom, cat. #FAB1561P, RRID: AB_10971948); VioBlue‐conjugated anti‐CD9 (Miltenyi Biotec, Lund, Sweden, cat. #130‐118‐809, RRID: AB_2733955) and APC‐conjugated anti‐CD73 (Thermo Fisher Scientific, cat. #17‐0739‐41, RRID: AB_1548701). Negative controls were prepared without any EV particles added. Staining was evaluated by an inverted epifluorescence microscope (Axio Observer 7, Zeiss, Oberkochen, Germany) using an oil‐immersed 63× objective lens. In total, 40 images were analysed for each sample (except for the negative control samples where eight images were analysed) and processed by Zen Blue 3.0 software (Zeiss). The mean number of EVs per image in the individual samples were measured in one technical replica.

### 2.12 Bioinformatics Analysis of Extracellular Vesicles and Cell Extract Mass Spectrometry Profiling Data

The raw protein abundance values of the MS data corresponding to any given protein was included in the analyses if expressed in all three biological replicates. For the label‐free quantification data of H1975 and H1975/OR EVs, the raw protein abundance values were used in subsequent analysis. For the TMTpro‐based quantification of H1975 and H1975/OR cell extracts, the raw protein abundance values were normalized (see **Supporting Information (S1)** for details). To determine the relative protein expression in the EVs MS data, statistical analysis was performed in Excel (V. 2019, Microsoft Office, Redmond, WA, USA). Fold change and *p* values (*t* test: *p* ≤ 0.05) were plotted in volcano plots using GraphPad Prism software v. 10.0.2 (GraphPad Software, Inc., LA Jolla, San Diego, CA, USA). The relative protein expression levels in either cells or EVs were also plotted as bar charts in GraphPad Prism software, using one‐way ANOVA (*p* < 0.05) for statistical verification.

The raw protein abundance values in the MS data of the EVs were sorted by cell origin (H1975 vs. H1975/OR) or treatment (no treatment vs. osimertinib) using the Qlucore Omics Explorer 3.9 software (Qlucore AB, Lund, Sweden) with a cutoff of *p* ≤ 0.01 and *q* ≤ 0.1. Protein scoring was also performed using machine learning and classifiers: K‐nearest neighbour (KNN), Support vector machine (SVM), RandomTrees and extreme gradient boosting (XGBoost), provided in the Qlucore Omics Explorer 3.9 software.

In silico StringDB analysis (www.string‐db.org (Szklarczyk et al. 2023)) of the proteins revealed by the Qlucore Omics Explorer 3.9 software, were used to suggest protein networks. Possible protein–protein interactions, biological processes and molecular function related processes were determined by the gene ontology (GO) analysis tool on the same platform. In brief, the protein expressions acquired by MS were sorted by fold change to compare the relative expression between H1975‐EVs versus H1975/OR‐EVs or Ptn. #3‐EVs versus Ptn. #5‐EVs. Pathways in which proteins were more frequently found in the H1975/OR‐EVs were compared against pathways obtained from Ptn. #3 and Ptn. #5 EVs data. The Gene Ontology (GO) and Kyoto Encyclopaedia of Genes and Genomes (KEGG) analysis was performed using R‐Studio (ver. 2024.12.0 Build 467) with the libraries: clusterProfiler (Wu et al. 2021) and Org. Hs. eg. db (Bioconductor R package, ver. 3.20). The plot was generated with the libraries: igraph (R package, ver. 2.1.4.9024), ggplot2 (Wickham et al. 2016), graph (R packager, ver. 2.2.1.9000) and reshape2 (Wickham 2007).

### 2.13 Bioinformatic Analysis of Proximity Extension Assay Data

The Qlucore Omics Explorer 3.9 software was also applied on the PEA profiling data of the serum‐isolated EVs. For the analyses related to PD‐L1 or CD73/NT5E expression in EVs, all samples were analysed, resulting in inclusion of some samples with EV protein NPX values below RIPA negative control if 60% of the samples in the total cohort expressed the protein over the negative control. The NPX values from the assay were applied and the association with PD‐L1 or CD73/NT5E expression was analysed by a rank regression tool within the Qlucore Omics Explorer 3.9 software. For analysis of protein expression in relation to best response, one patient (Ptn. #7 with PD) was excluded as this was the only patient that did not respond to treatment and displayed PD. Further analyses were done using single EV sample protein NPX values using two‐group Mann–Whitney in the GraphPad Prism software v. 10.0.2.

### 2.14 Flow Cytometry Profiling of Chondroitin Sulfate Proteoglycan 4 Cell Surface Expression

The cell surface expression of chondroitin sulfate proteoglycan 4 (CSPG4) was studied on H1975, H1975/OR, HCC827 or HCC827/OR cells prior‐ and post‐treatment with 0.1 µM osimertinib for 48 h. The cells were trypsinized and fixed in 4% formaldehyde solution (Bioreages, Ellös, Sweden) before staining with a FITC‐conjugated CSPG4 antibody (Invitrogen eBioscience, cat. #53‐6504‐82, RRID: AB_10853964) targeting an extracellular epitope, diluted 1:50 in PBS. The staining of the cells was analysed on a NovoCyte 3000 flow cytometer. The median fluorescence intensity of the 12,000 captured events was quantified and the autofluorescence of the cells was assessed by including an unstained sample of each cell type.

### 2.15 Chondroitin Sulfate Proteoglycan 4 siRNA Transfection and Assessment of Cell Viability

To evaluate the effect of inhibition of chondroitin sulfate proteoglycan 4 (CSPG4) expression, H1975/OR cells were transiently transfected with siRNA targeting CSPG4 mRNA (Thermo Fisher Scientific, cat. AM16708, ID:146145, RRID:SCR_008452) or non‐targeting siRNA (Stealth RNAi siRNA Negative Control, Invitrogen, cat. 12935300) per manufacturer's instructions using DharmaFECT 1 transfection reagent (Thermo Fisher Scientific, cat. T‐2001‐01/ DHART‐2001‐02). Cells were seeded in six‐well plates at a density of 250,000 cells per well per treatment. The next day, 50 nM of either siRNA was added to the cells in serum‐free RPMI‐1640 media supplemented with 2 mM l‐glutamine. To assess the effect of CSPG4 inhibition on osimertinib induced cell toxicity, 0.1 µM osimertinib was added at the same time as the transfection reagents. Cell viability was assessed after 48 h using trypan blue exclusion staining and manual counting. Treated cells were compared to untreated cells set to 100% and analysed by One‐way Anova. The knock‐down of CSPG4 mRNA expression was evaluated 24 h post transfection. RNA was isolated from a cell pellet by the RNeasy kit (Qiagen, cat. 74104) and reverse transcribed into cDNA using the High‐capacity reverse transcription kit (Thermo Fisher Scientific, cat. #4374966). The program applied was 25°C, 10 min, 37°C, 1 h, 95°C for 5 min. CSPG4 mRNA expression was analysed by real time quantitative PCR in which 1 µL of cDNA was added to Taqman fast advanced mastermix (cat. 4444557, Applied Biosystems / Thermo Fisher Scientific) and Taqman FAM‐labelled primer/probe mix for CSPG4 (Hs05636647_s1; Thermo Fisher Scientific, cat. #4331182) or GAPDH (Hs02758991_g1; Thermo Fisher Scientific). Real time PCR was performed on a Bio‐Rad CFX96 (Model C1000 Touch Thermal Cycle) with the following program: 20 s at 95°C, 45 cycles of 95°C for 1 s and 20 s at 60°C. CSPG4 mRNA level was calculated by the 2^−ΔCt^ formula and these values were after GAPDH normalization applied to give the relative expression in the different samples.

## 3 Results

### 3.1 Mass Spectrometry Profiling of Extracellular Vesicles Reveals Proteins Associated With Osimertinib Resistance in Mutant EGFR Driven Non‐Small Cell Lung Cell Lines

**FIGURE 1:**
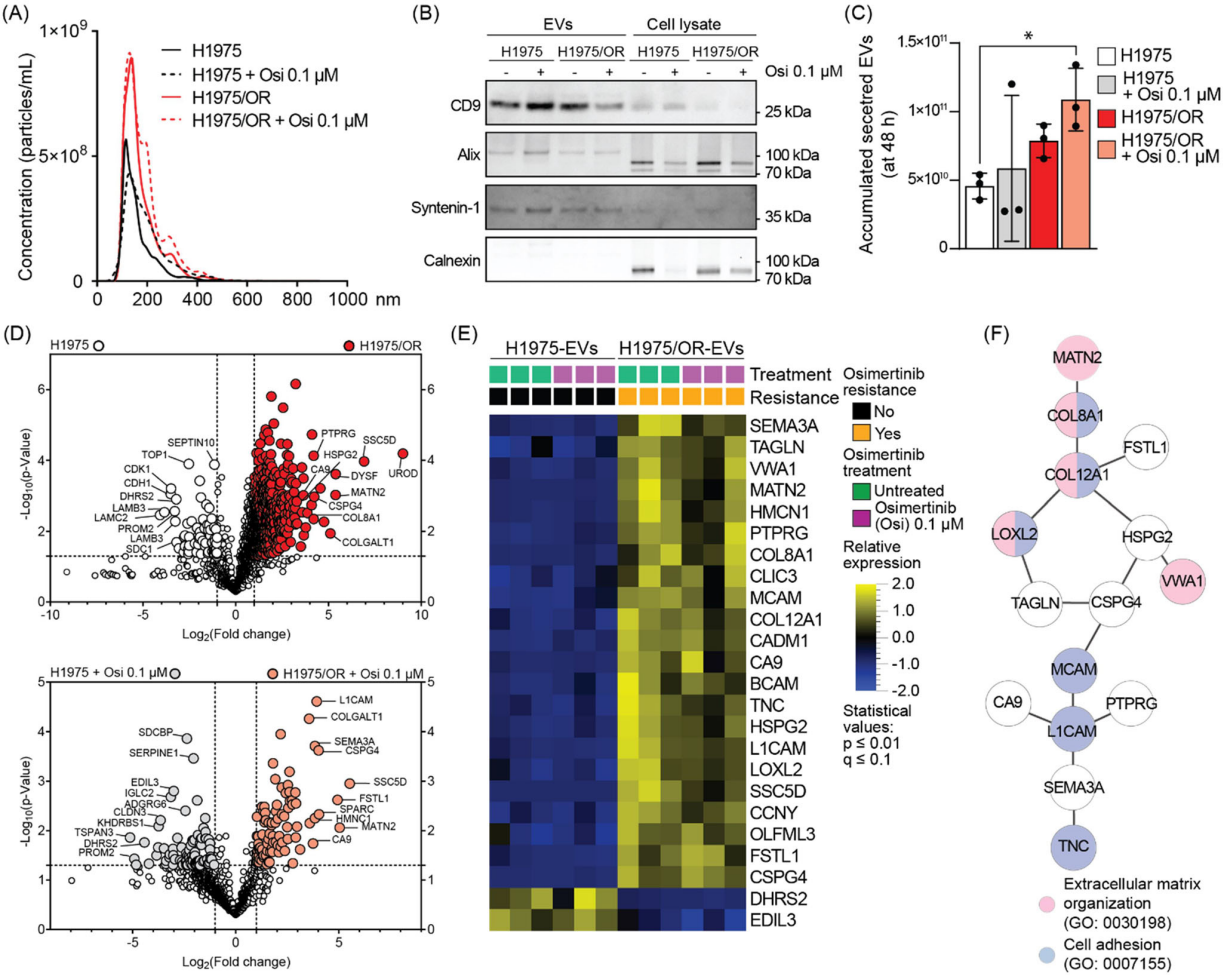
**Mass spectrometry profiling of extracellular vesicles to reveal putative osimertinib resistance associated networks in mutant *EGFR* driven non‐small cell lung cancer cells**. **(A)** The size distribution of extracellular vesicles (EVs) isolated from H1975 or H1975/OR cell culture media before and after osimertinib exposure for 48 h are presented with respect to particle size (in nm) and concentration (particles/mL). Average data from three biological replicates is shown. **(B)** EVs and cell extracts were studied by western blot analysis for expression of CD9, alix and syntenin‐1. Calnexin was used to assess general cellular protein contamination in the EVs samples. Five microgram of protein was loaded from each sample (EVs or cell lysate). **(C)** The total number of EVs at the time of harvest (48 h post osimertinib treatment) was calculated in three independent biological experiments. One‐way Anova, ∗; *p* value < 0.05 versus H1975. **(D)** Volcano plots depicting the mass spectrometry (MS) based protein raw expression values in EVs released from H1975 and H1975/OR cells to cell culture media prior (***top panel***) or post osimertinib (***bottom panel***) treatment. The data are based on the mean of three biological replicates. *T*‐test, cutoff: *p* ≤ 0.05. **(E)** Protein signatures were filtered out from the MS data using Qlucore software, comparing EVs released from H1975/OR cells or H1975 cells pre‐ and post‐osimertinib treatment. Data are based on three biological replicates. **(F)** Representation of the obtained StringDB analysis signature of the top proteins found in the EVs from H1975/OR cells presented in **(E)**.

We focused on extracellular vesicles (EVs) to identify proteins associated with osimertinib refractoriness in the *EGFR*‐mutant responsive H1975 or refractory H1975/OR cell lines, respectively (Figure S1A). Assessment of osimertinib cytotoxicity confirmed that H1975/OR cells were osimertinib refractory relative to the parental H1975 cells (Figure S1B‐D). The EVs which were isolated from the culture media of these cells had mode sizes ranging between 118 and 133 nm (Figure 1A). The EV morphology was confirmed by transmission electron microscopy (TEM) (Figure S1E). The International Society for Extracellular Vesicles (ISEV) has suggested a set of markers to be enriched in EVs (Welsh et al. 2024), and here the expression of the tetraspanin CD9, the ESCRT pathway related protein alix, and the syndecan family protein syntenin‐1 were all expressed in the SEC‐isolated EVs samples, while calnexin expression was not detected (Figure 1B). Analyses of the accumulated number of EVs released into the cell culture media suggested a trend toward higher EV release from the H1975/OR cells relative to H1975 cells, with a further increase observed after osimertinib treatment (*p* value < 0.05) (Figure 1C).

Mass spectrometry (MS) based protein profiling was conducted on EVs isolated from the H1975 and H1975/OR cells prior and post treatment with osimertinib for 48 h. The analysis revealed that 593 proteins exhibited a significantly higher expression (*p* value ≤ 0.05) in EVs isolated from media of untreated H1975/OR cells as compared to EVs from their untreated parental H1975 cells (Figure 1D **, top panel**). When assessing the protein expression after osimertinib treatment (0.1 µM), 90 proteins had a higher expression in EVs from media of H1975/OR cells, in comparison to the 89 proteins which displayed higher expression in the EVs from media of H1975 cells (*p* value ≤ 0.05) (Figure 1D **, bottom panel**). Gene set enrichment analysis revealed that a proportion of the identified proteins in the H1975/OR EVs were mapped to the KEGG focal adhesion and proteoglycans in cancer pathways (Figure S2A). Interestingly, both these pathways had more protein identities that had a higher expression in the H1975/OR EVs as compared to H1975 EVs (Figure S2B‐C).

A signature of 24 proteins associated with osimertinib resistance was filtered out using Qlucore bioinformatics (*t*‐test, two group, *p* value ≤ 0.01) and further explored using StringDB analysis (Figure 1E,F, Table S1). Results showed a core of proteins, for example, chondroitin sulfate proteoglycan 4 (CSPG4), heparan sulfate proteoglycan 2 (HSPG2), transgelin (TAGLN), lysyl oxidase homolog 2 (LOXL2), collagen alpha‐1(XII) chain (COL12A1), von Willebrand factor A domain containing 1 (VWA1) and matrilin 2 (MATN2) (Figure 1F). The proposed underlying biological pathways of this network were primarily extracellular matrix organization (GO: 0030198) and cell adhesion (GO: 0007155). To further validate this putative osimertinib resistance signature in silico, different machine learning algorithms (KNN, Random Trees, SVM and XGBoost) included in the Qlucore software were applied on the same entire MS‐data set from the EVs. The analysis revealed a signature including CSPG4, cell adhesion molecule 1 (CADM1), cyclin y (CCNY), follistain‐related protein 1 (FSTL1), COL12A1, neural cell adhesion molecule L1 (L1CAM), VWA1, cell surface glycoprotein MUC18 (MCAM), receptor‐type tyrosine‐protein phosphatase gamma (PTPRG) and hemicentin 1 (HMNC1) (Table S2).

### 3.2 Extracellular Vesicle‐Expressed Chondroitin Sulfate Proteoglycan 4 Shows Association to Osimertinib Resistance

**FIGURE 2:**
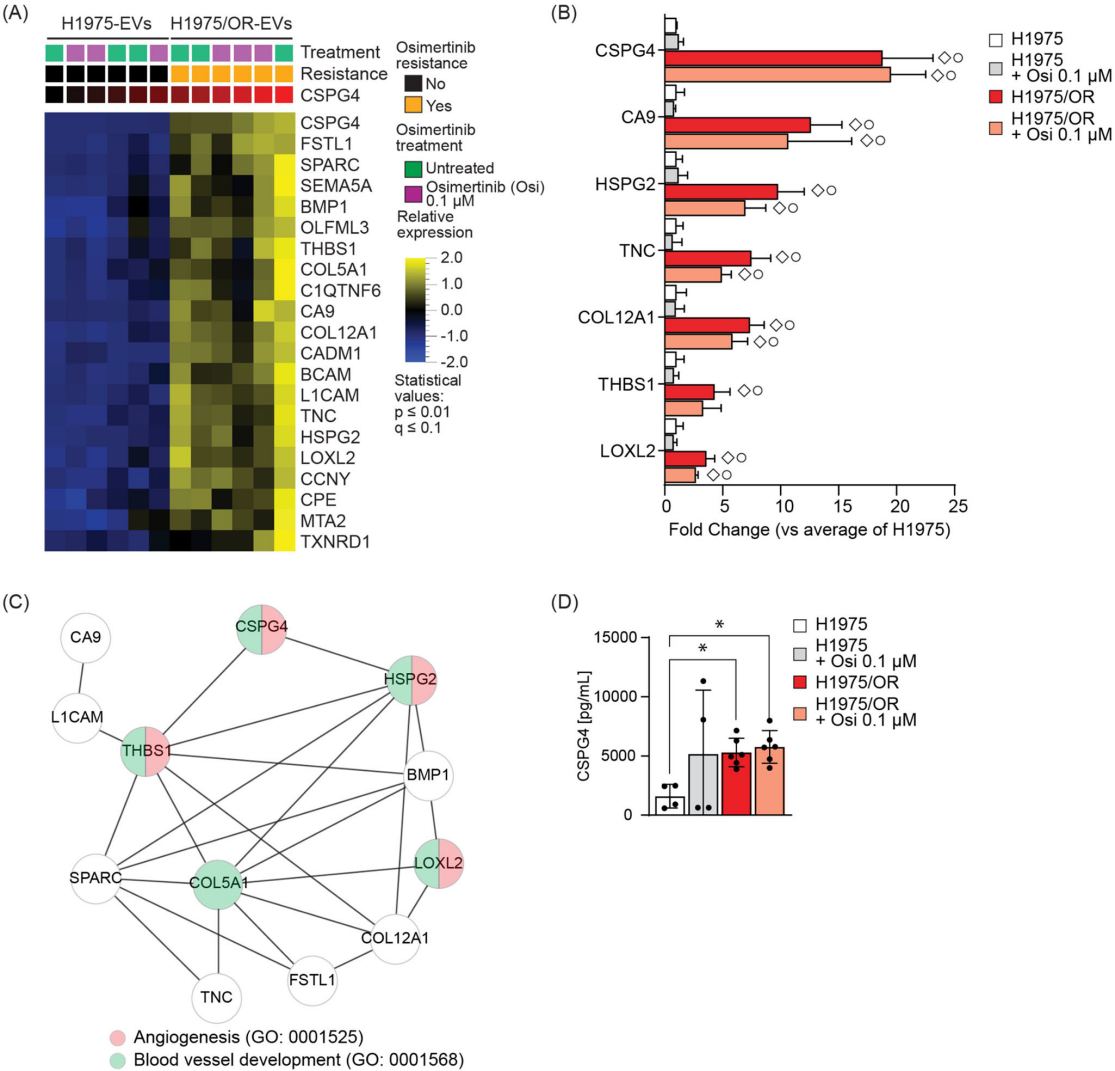
**Extracellular vesicles isolated from mutant *EGFR* driven non‐small cell lung cancer cell lines express chondroitin sulfate proteoglycan 4**. **(A)** A protein signature associated with chondroitin sulfate proteoglycan 4 (CSPG4) expression in the extracellular vesicles (EVs) released from H1975 or H1975/OR cells prior or post osimertinib is shown. The signature was obtained by rank regression analysis of the MS protein expression data, using the Qlucore platform with a *p* value ≤ 0.01 and a *q* value ≤ 0.1. The CSPG4 expression range is given from low (black) to high (red). For the original MS count data of the proteins see Table S1. **(B)** The fold change in average protein abundance is shown for EVs from H1975 or H1975/OR cell culture media prior and post osimertinib (0.1 µM; 48 h). Data are from three biological replicates. Two‐way Anova, ◇ *p* value ≤ 0.05 versus H1975, ◯ *p* value ≤ 0.05 versus H1975 + Osi 0.1 µM. **(C)** Representation of the StringDB analysis of selected proteins from the rank regression analysis of CSPG4 shown in panel **B**. **(D)** Protein quantification of CSPG4 by ELISA on non‐lysed EVs from untreated H1975 cell culture media (1305 ± 986 pg/mL), after osimertinib (0.1 µM; 48 h, 6668 ± 5483 pg/mL), from untreated H1975/OR cell culture media (5549 ± 1420 pg/mL) or after osimertinib (0.1 µM; 48 h; 5914 ± 2006 pg/mL) are presented from four biological replicates for H1975 and six biological replicates for H1975/OR. One‐way Anova, ∗; *p* value ≤ 0.05 versus H1975.

Chondroitin sulfate proteoglycan 4 (CSPG4) has been demonstrated to regulate cell migration, and to function as a scaffold protein for both RTKs and FAK signalling networks in malignant melanoma (Price et al. 2011; Yang et al. 2019; Yang et al. 2004), breast cancer (Wang et al. 2010) and sarcoma (Cattaruzza et al. 2013). CSPG4 has also been proposed and evaluated as a treatment target as well as for imaging purposes in various cancers, using antibody‐based approaches (Nicolosi et al. 2015; Koopmans et al. 2019; Ilieva et al. 2017; Teppert et al. 2023). Yet, the role of CSPG4 in NSCLC has, to our knowledge, not been extensively studied and therefore, we focused on proteins associated with CSPG4 expression in our MS protein profiling data of the EVs (Figure 2A). A statistically significant (*p* value ≤ 0.01, *q* value ≤ 0.1) associated signature which consisted of 20 proteins, including HSPG2, LOXL2, TNC, CA9, COL12A1 and FSTL1, among others was revealed (Figure 2A, Table S1). Evaluating some of these proteins individually, that are, CSPG4, CA9, HSPG2, TNC, COL12A1, THBS1 and LOXL2, confirmed that their expression was significantly higher in EVs from cell culture media of H1975/OR cells as compared to EVs from H1975 cell media (two‐way Anova, *p* value ≤ 0.05; Figure 2B). Next, a StringDB analysis was performed, which revealed a CSPG4 associated cluster to be linked to angiogenic related processes (GO:0001525, THBS1, HSPG2, CSPG4, LOXL2) and blood vessel development (GO:0001568, COL5A1, THBS1, HSPG2, CSPG4, LOXL2) (Figure 2C).

CSPG4 expression on non‐lysed EVs was also analysed by ELISA (Figure 2D). A significantly higher level of CSPG4 (3.3‐fold, two‐way Anova, *p* value ≤ 0.05) was found on the EVs isolated from the cell culture media of H1975/OR cells relative those from the parental H1975 cells, while no differences in CSPG4 levels on EVs were evident after osimertinib treatment. We also analysed CSPG4 expression on EVs isolated from another *EGFR*‐mutant osimertinib resistant NSCLC cell line, HCC827/OR, by ELISA and observed a clear expression, albeit no difference of CSPG4 expression prior and post osimertinib treatment was seen (Figure S3).

### 3.3 Chondroitin Sulfate Proteoglycan 4 is Expressed in Non‐Small Cell Lung Cancer Cells Driven by Mutated EGFR

**FIGURE 3:**
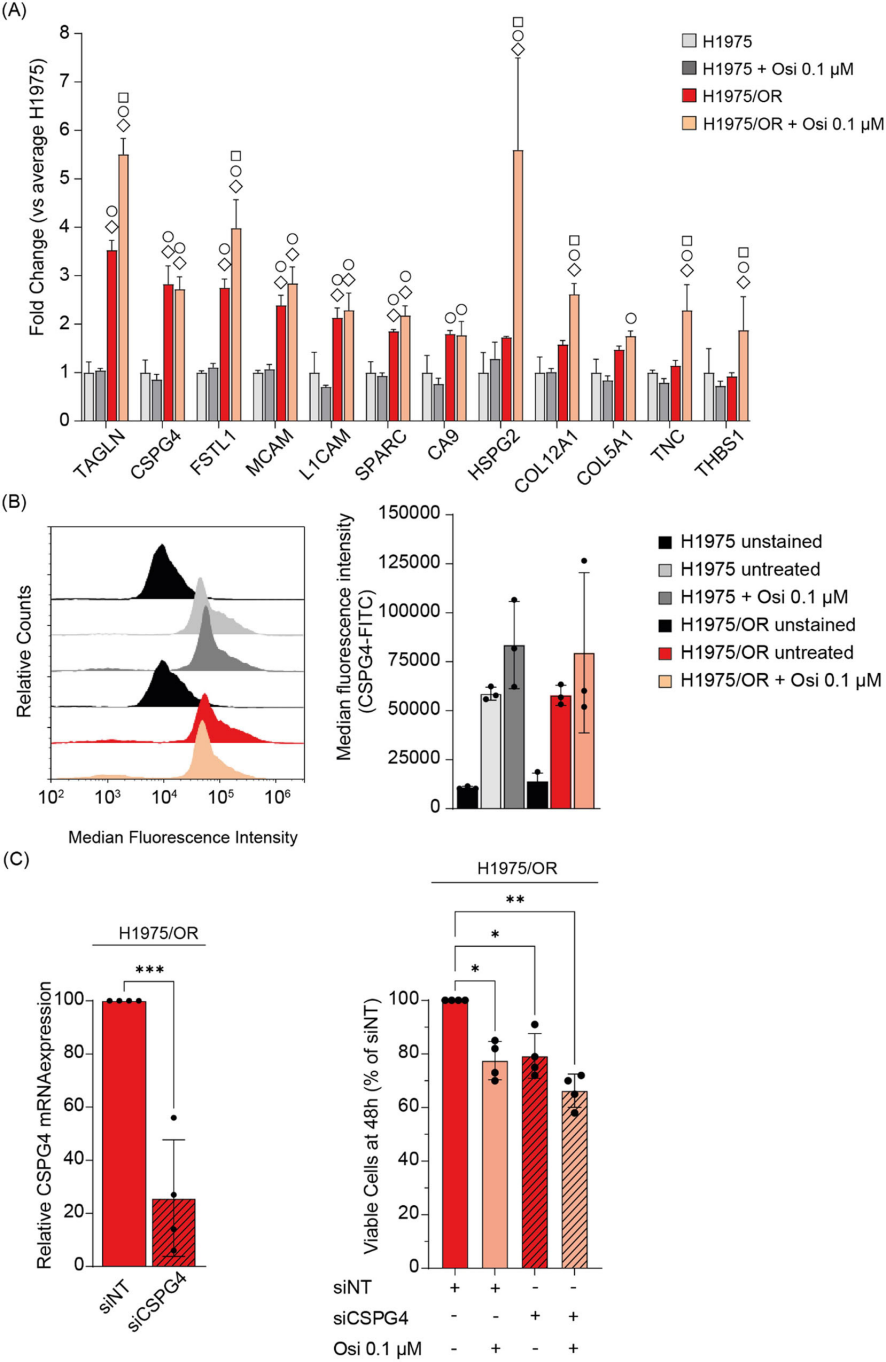
**Chondroitin sulfate proteoglycan 4 is expressed on mutant *EGFR* expressing non‐small cell lung cancer cells and influences their cell viability**. **(A)** Bar chart showing expression levels of the osimertinib refractoriness EV associated proteins in TMT pro‐labelled H1975 and H1975/OR cell extracts prior‐ and post osimertinib (0.1 µM; 48 h) as revealed by mass spectrometry (MS) quantification. The fold change in mean protein abundance is shown in H1975/OR versus H1975 cell extracts. Data from three biological replicates is shown. Two‐way Anova, ◇ *p* value ≤ 0.05 versus H1975, ◯ *p* value ≤ 0.05 versus H1975 + Osi 0.1 µM, ◻ *p* value ≤ 0.05 versus H1975/OR. For data normalization, see **Supporting Information (S1)**. **(B)** H1975 or H1975/OR cells were stained with a FITC‐conjugated CSPG4 antibody targeting an extracellular epitope. **Left panel**: Histograms of CSPG4‐FITC staining from one replicate are shown, where unstained correspond to cells without addition of the CSPG4‐FITC antibody. **Right panel**: Quantification of the FITC median fluorescence intensity. Data are based on three experiments. No statistical difference was found by multiple comparison testing (one‐way Anova) when comparing the median fluorescent intensities of the untreated and treated cells, or when comparing the two cell lines. **(C)** H1975/OR cells were transiently transfected with siRNA targeting CSPG4 (siCSPG4) or non‐targeting siRNA (siNT) alone or combined with osimertinib (0.1 µM). **Left panel**: Real time quantitative PCR assessment of CSPG4 mRNA expression at 24 h post siRNA transfection. Data shown are from four biological experiments, with significance analysed by *t* test, ***; *p* ≤ 0.001. **Right panel**: Cell viability was assessed at 48 h post transfection by trypan blue counting. The number of viable cells from four biological replicates are shown in percentage relative to the respective siNT controls. One‐way Anova was performed, *; *p* value ≤ 0.05, **; *p* value ≤ 0.01.

The EVs protein signature found to be associated with osimertinib resistance (Figures 1F and 2C) was subsequently studied in H1975 and H1975/OR cell extracts by MS using tandem mass tag (TMT)‐pro labelling (Figure 3A). The proteins CSPG4, HSPG2, MCAM, L1CAM and TAGLN were similar as in EVs, found to have a significantly elevated expression in the osimertinib resistant H1975/OR cells. In contrast, TNC and THBS1 only exhibited a significantly higher expression in H1975/OR cells after osimertinib treatment (approximately 2‐ and 1.5‐fold, respectively, (two‐way Anova, *p* value ≤ 0.05 vs. H1975) (Figure 3A)). To verify the expression of CSPG4 on the surface of the H1975 or H1975/OR cells, flow cytometry was performed on fixed, non‐permeabilized cells (Figure 3B). CSPG4 expression on the cell surface was evident both prior and post osimertinib treatment. However, in contrast to what was seen in the total protein expression profiling by MS, the surface expression levels were found to be similar between the cell lines (Figure 3B). The CSPG4 expression was also examined in another pair of *EGFR*‐mutant cell lines, HCC827 and HCC827/OR, before and after osimertinib treatment (Figure S4). Although all the cells exhibited a clear CSPG4 surface expression, no statistical difference in CSPG4 levels was evident between HCC827 and HCC827/OR untreated cells or post osimertinib.

Next, we studied if the osimertinib refractory H1975/OR cells were dependent on CSPG4 expression for cell survival. For this purpose, we suppressed CSPG4 expression through transient siRNA transfection (siCSPG4). After 24 h, the residual CSPG4 mRNA levels were about 25% as compared to the non‐targeting (siNT) control (*t*‐test, *p* ≤ 0.001) as measured by qPCR (Figure 3C **, left panel**). The cell viability was statistically significant reduced by approximately 20% after 48 h of siCSPG4 transfection as compared to the siNT control, thereby providing a similar effect as obtained by 0.1 µM osimertinib (one‐way Anova, *p* value ≤ 0.05) (Figure 3C **, right panel**). Moreover, combining siCSPG4 with 0.1 µM osimertinib decreased the cell viability by around 30% as compared to the siNT control, suggesting a slight additive effect. Taken together, our results show that mutant *EGFR*‐driven NSCLC cells made resistant to osimertinib have increased CSPG4 expression and reducing its expression by transient siRNA transfection had a modest, but still significant, effect on the cell viability, illustrating a possible role for CSPG4 in osimertinib refractoriness at least in a subset of mutant EGFR‐driven NSCLC.

### 3.4 Molecular and Clinical Characteristics of the Non‐Small Cell Lung Cancer Cohort

To further explore proteins expressed in EVs in the context of osimertinib response, we profiled serum‐isolated EVs from patients included at Karolinska University Hospital in the phase II clinical trial TREM (NCT02504346) evaluating osimertinib in second‐line (Eide et al. 2020). There was no selection of patients, as all patients with samples available at the Karolinska Hospital were included (total no. patient included: 21; sample at baseline and progression: 17; sample only at progression: 4). All had stage IV adenocarcinoma with different activating *EGFR* mutations at the time of study inclusion (Table 1). Of note, out of the patients analysed in this work, 16 patients had an *EGFR T790M* positive tumour. The patients had been given first‐ or second‐generation EGFR‐TKIs prior to osimertinib and accordingly the response was heterogeneous in terms of magnitude and duration (Table 1). Evaluation of the best treatment response showed that 12 patients reached partial response (PR), eight patients had stable disease (SD), and one patient progressive disease (PD). The progression‐free survival (PFS) of the patients ranged from 1.6 to 25.2 months with a median of 9.1 months. The PFS in this cohort were in the same range as in the entire TREM cohort (Eide et al. 2020) (9.1 vs. 8.9 months, respectively). The overall survival (OS) spanned 7.5–51.1 months with a median of 22.9 months which was longer compared to the total TREM cohort (17.9 months, [Table 1, Eide et al. 2020]).

The mean values of the analyzed mode sizes of the serum‐isolated EVs from the patients were 92 nm at baseline and 96 nm at progression (Figure S5B). The EV concentration in the serum at baseline ranged between 2 × 10^10^ and 1 × 10^11^ (Figure S5C, ***left panel***) and between 2 × 10^10^ and 9 × 10^10^ EVs/mL at progression (Figure S5C **, right panel**). Western blot analysis confirmed expression of tetraspanin CD9, alix and flotillin‐1 in the serum‐isolated EVs (Figure S5D). TEM assessment of the isolated particles from serum of two of the patients at baseline (Ptn. #4 and Ptn. #8) verified EV size and morphology (Figure S5E).

### 3.5 Chondroitin Sulfate Proteoglycan 4 is Expressed on Extracellular Vesicles From Serum of Non‐Small Cell Lung Cancer Patients

**FIGURE 4:**
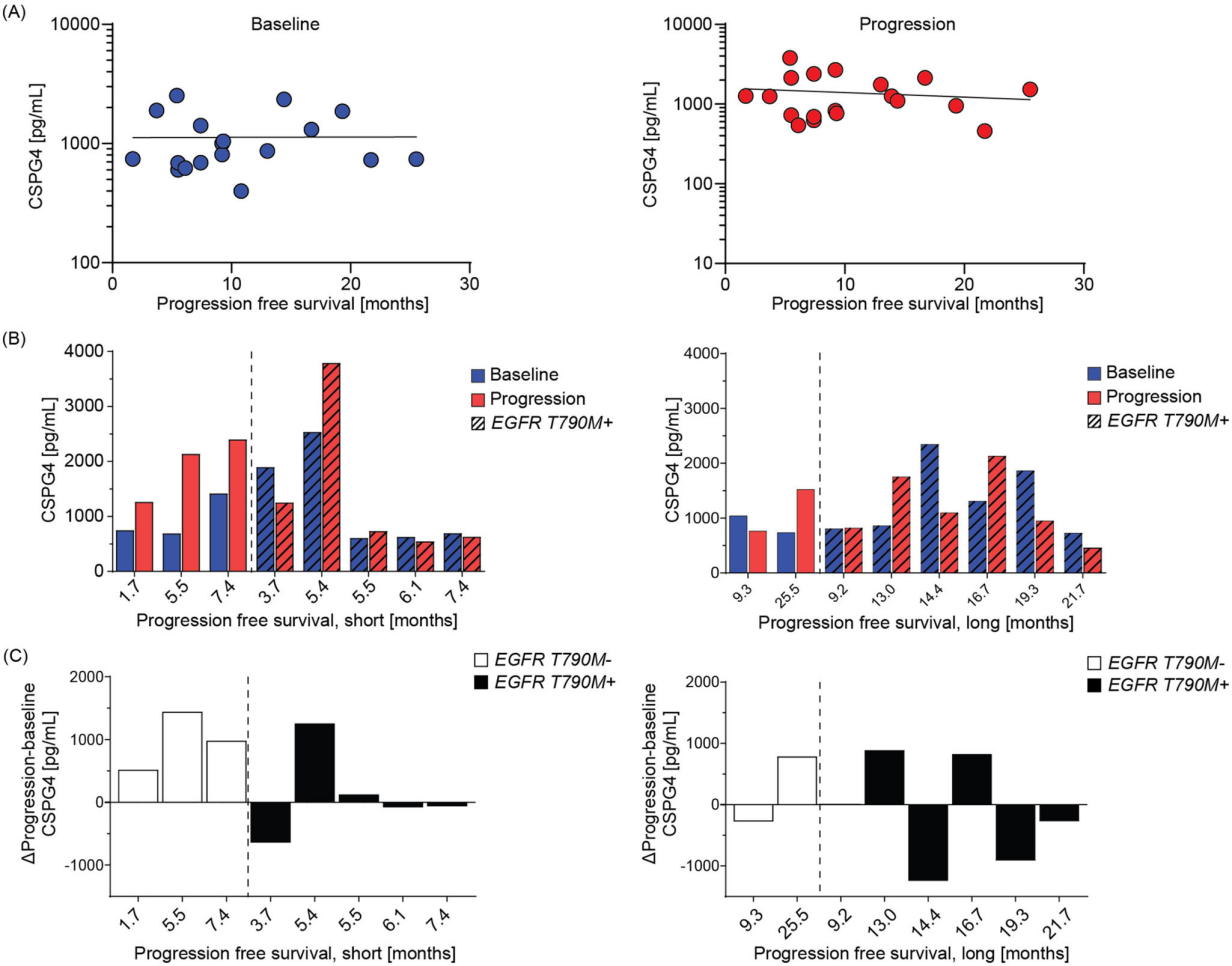
**Expression of chondroitin sulfate proteoglycan 4 on extracellular vesicles isolated from serum of non‐small cell lung cancer patients**. Chondroitin sulfate proteoglycan 4 (CSPG4) expression on extracellular vesicles (EVs) isolated from serum of the non‐small cell lung cancer patient cohort (Table 1) was studied by enzyme‐linked immunosorbent assay (ELISA). The CSPG4 concentrations in the samples were all within the standard curve of the assay. **(A)** CSPG4 (pg/mL) on EVs at baseline (***Left panel***) or at progression (***Right panel***) were analysed in relation to patients´ progression‐free survival (PFS). Linear regression revealed no significance. **(B)** CSPG4 expression on EVs were studied in relation to PFS divided into PFS <8.9 months> as reported by (Eide et al. 2020), delimited by *EGFR T790M* mutation (dotted line). **(C)** The difference in CSPG4 expression levels on the EVs at baseline and progression of each patient is presented in relation to PFS (as described in **(B)** and delimited by *EGFR T790M* mutation (no: white bars, yes: black bars)).

Given the association between CSPG4 expression in EVs of the mutated *EGFR*‐driven NSCLC cells in vitro and osimertinib refractoriness, we next evaluated CSPG4 levels on serum‐isolated EVs of the NSCLC patient cohort at baseline and progression (Table 1). We for the first time report that CSPG4 is expressed on EVs isolated from serum of NSCLC patients, with expression evident both at baseline and at progression (Figure 4A,B). However, the EVs CSPG4 expression at either time point was not found to significantly correlate with PFS (*p* value > 0.05) or OS (*p* value > 0.05) (Figures 4A and S6A). Moreover, there was no significant difference in CSPG4 expression in EVs isolated from serum of patients with *EGFR T790M* positive or negative tumours at study inclusion (Figures 4B and S6B), nor was there a significant change in CSPG4 expression levels between baseline and progression that could be linked to the individual patient outcome, that is, PFS or OS (Figures 4C and S6C). In summary, CSPG4 expression was evident in the EVs from patients with *EGFR*‐mutant NSCLC. However, in this patient cohort, where prior lines of EGFR‐TKIs had been given, an association with patient outcomes was not found.

### 3.6 A Protein Signature Associated With Osimertinib Resistance is Found in Extracellular Vesicles Isolated From Serum of Non‐Small Cell Cancer Patients

To further explore the serum‐isolated EVs protein cargo, MS analysis was applied on EVs isolated from serum of two patients (Ptn. #3 and #5) with short or long PFS (5.5 vs. 16.5 months) or OS (9.4 vs. 37.1 months), respectively. Both patients had PR as the best treatment response.The identified proteins were analyzed against the vesiclepedia top 100 list with proteins often identified in EVs. 33 of these proteins were expressed in all samples and up to 42 proteins expressed in at least two samples (Figure S7). We compared the MS‐identified proteome profile of the EVs from Ptn. #3 and Ptn. #5 at baseline. The analysis revealed that 208 proteins were shared and had no significant differences in expression between the samples, while 115 proteins and 37 proteins had a higher expression in Ptn. #3 and Ptn. #5, respectively (Figure S8A, Table S3).

To reveal if there was a signature of proteins in the EVs that were associated with osimertinib resistance (Figure 1), the protein identities of EVs from Ptn. #3 (short PFS and OS) were compared to protein in EVs released from H1975/OR cells to cell culture media (Figure S8B, Table S3). Results showed an overlap of 49 proteins and among these COL12A1, HSPG2, TAGLN and TNC were found, all which were associated with an osimertinib refractory phenotype of the H1975/OR cells and their released EVs (Figures 2 and 3). When the same analysis was made between Ptn. #5, who had a better outcome on osimertinib, and H1975/OR derived EVs, four proteins were in common, out of which only VWA1 was linked to an osimertinib refractory phenotype (Figure S8C, Table S3). Furthermore, in silico GO and KEGG pathway analysis showed that several pathways were shared between EVs from H1975/OR cells and Ptn. #3 as presented in Figure S8D. In summary, our analyses highlight multiple proteins in EVs that link to an osimertinib refractory phenotype which warrant further analyses.

### 3.7 Serum‐isolated extracellular vesicles of non‐small cell lung cancer patients express protein signatures associated with program cell death ligand‐1 and cluster of differentiation CD73/5’‐nucleotidase

To further reveal protein signatures in the EVs associated with an osimertinib responsive or refractory phenotype, we next focused on immune signalling components. As these are difficult to reveal by global MS analysis due to their rather low expression levels, we applied proximity extension assay (PEA); a multiplex antibody‐based assay with high specificity as well as high sensitivity (Assarsson et al. 2014; Lundberg et al. 2011) that previously has been used for protein profiling of EVs (Larssen et al. 2017; Indira Chandran et al. 2019).

**FIGURE 5:**
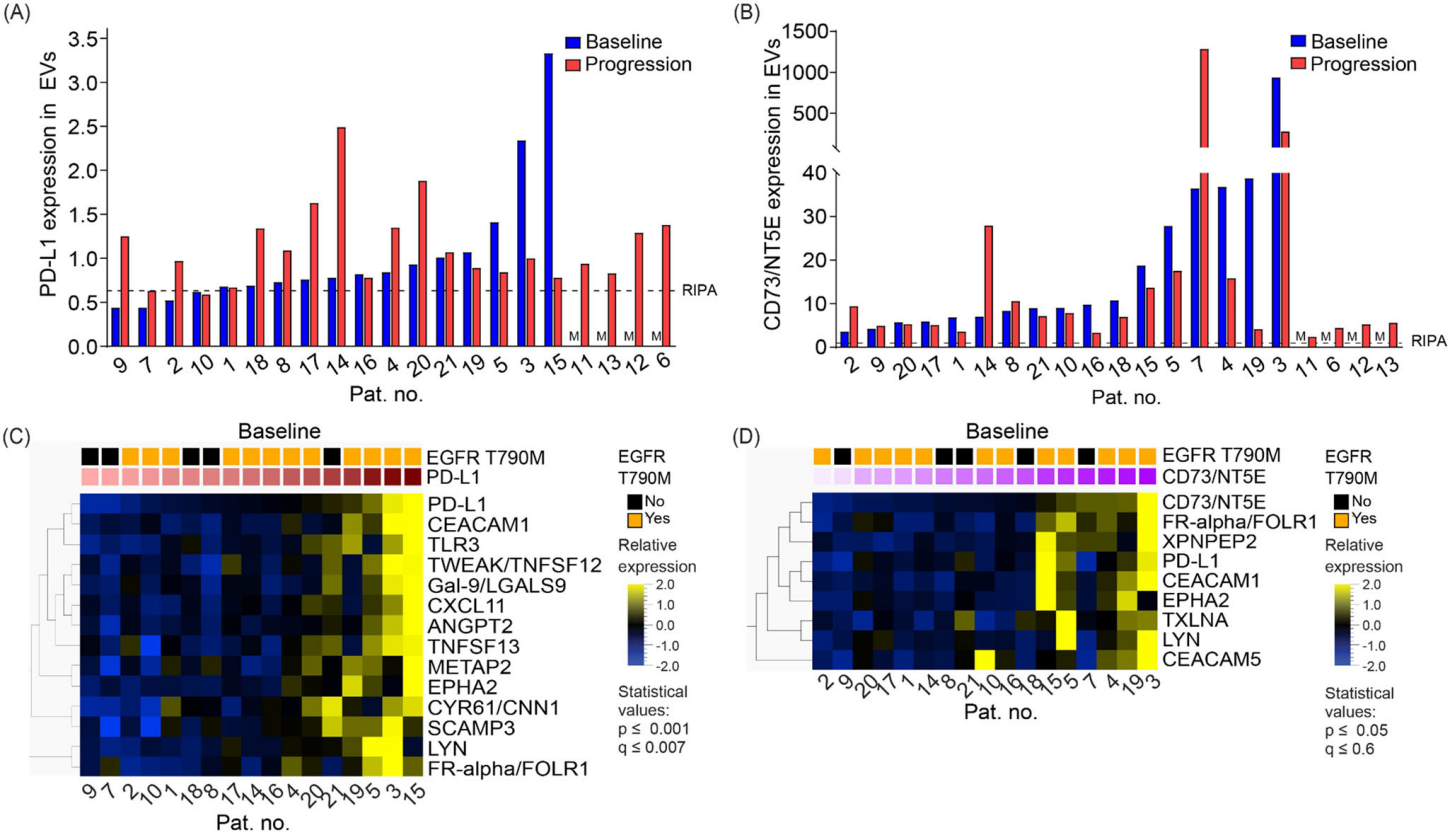
**Extracellular vesicles isolated from serum of non‐small cell lung cancer patients express PD‐L1 and CD73/NT5E immune regulatory proteins**. Extracellular vesicles (EVs) isolated from serum of non‐small cell lung cancer patients taken at baseline prior to osimertinib treatment or at progression were analysed (Table 1). The protein cargo of the EVs were studied by proximity extension assay (PEA) on the Oncology II and Immuno‐oncology panels with the protein expression values (presented as NPX) further analysed by the Qlucore software with indicated statistics. Data presented were obtained from EVs isolated from one replicate of each serum sample. Note that baseline samples were missing for four patients (Ptn. #6, #11, #12, #13) giving a total of 17 samples. **(A)** PD‐L1 expression or **(B)** CD73/NT5E expression values in EVs from the individual patient samples were linearized from the NPX values of the PEA assay. Data are presented in order of increasing expression of PD‐L1 or CD73/NT5E in the baseline samples, respectively. The dotted line represents the NPX values of the RIPA negative control. The ‘M’ indicates missing baseline samples. **(C)** A protein signature associated with PD‐L1 expression in the serum EVs isolated at baseline was obtained by rank regression analysis of the PEA expression data using the Qlucore software with *p* value ≤ 0.001 and *q* value ≤ 0.007. The PD‐L1 expression ranges from low (light pink) to high (red). **(D)** A CD73/NT5E associated protein signature in EVs was sorted out as in **(C)** with *p* value ≤ 0.05 and *q* value ≤ 0.6). The CD73/NT5E expressions range from low (light purple) to high (dark purple).

Programmed cell death ligand‐1 (PD‐L1) (Holder et al. 2024; Zhang et al. 2024; Cheng et al. 2022) and cluster of differentiation 73/5'‐nucleotidase (CD73/NT5E) (Kowash and Akbay 2023; Passarelli et al. 2019) both play critical roles in the tumour‐immune system interplay. Importantly, an increased CD73/NT5E expression has been shown in mutant‐*EGFR* positive tumours and been linked to a less favourable outcome (Ferrara et al. 2022; Kim et al. 2023; Le et al. 2021). Moreover, both these proteins were reported to be expressed in EVs isolated from plasma or serum of cancer patients, where they were found to regulate for example immune checkpoint inhibitor (ICI) response (Wu et al. 2021; De Miguel‑Perez et al. 2024; Turiello et al. 2022; Ploeg et al. 2021). Given these reports, we focused on the expression of CD73 and PD‐L1 and their associated signatures in the EVs isolated from serum of the NSCLC patients both at baseline and at progression. For these analyses, the EVs were lysed and subjected to PEA profiling combining two different panels, the Oncology II and Immuno‐Oncology, respectively. The experimental setup is shown in Figure S5A. Results revealed PD‐L1 expression in EVs at levels above the RIPA negative control in 13 out of 17 samples at baseline, and in 19 out of the 21 samples at progression (Figures 5A and S9A). The PD‐L1 expression in the serum‐isolated EVs was further confirmed by immunofluorescence‐based single EV analyses of intact EVs using CD9 for capturing as we previously described (Sahu et al. 2023; Stridfeldt et al. 2023) (Figure S9B). With respect to CD73/NT5E, PEA profiling of lysed EVs revealed a clear yet heterogeneous expression level both in the EVs isolated from baseline samples as well as at progression (Figure 5B). We also confirmed the expression of CD73/NT5E in EVs by ELISA (Figure S9C). In addition, the surface EV expression level of CD73/NT5E was verified by single EV fluorescence microscopy (Figure S9D).

To find proteins correlated with PD‐L1 expression in the EVs, we applied a rank regression analysis on the data from the EVs PEA profiling on samples taken at baseline using Qlucore bioinformatic software (Figure 5C). Our results showed that 13 proteins were statistically significantly (*p* value ≤ 0.001, *q* value ≤ 0.007) linked with the PD‐L1 expression pattern in the EVs and these were, among others, cellular communication network factor 1 (Cyr61/CCN1), carcinoembryonic antigen‐related cell adhesion molecule 1 (CEACAM1), toll‐like receptor 3 (TLR3), C‐X‐C motif chemokine 11 (CXCL11), secretory carrier‐associated membrane protein 3 (SCAMP3) and folate receptor alpha (FR‐alpha/FOLR1) (Figure 5C). None of these proteins were, however, linked to PD‐L1 expression in the progression samples (*p* value ≤ 0.05, *q* value ≤ 0.46) (Figure S9E).

We subsequently did a similar rank regression analysis in relation to CD73/NT5E expression levels in the EVs (Figure 5D **;** *p* value ≤ 0.05, *q* value ≤ 0.6). At baseline, eight proteins showed an association to CD73/NT5E, whereas at progression 18 proteins were linked (Figure S9F **;** *p* value ≤ 0.01, *q* value ≤ 0.06). Thus, our profiling of EVs in the context of PD‐L1 or CD73/NT5E expression patterns suggests that the effects of osimertinib on the immune signalling landscape (Kim et al. 2023; Le et al. 2021) can be detected in serum‐isolated EVs. However, as we studied total EVs in the serum samples, further studies are needed to understand the origin of these EVs with respect to different immune cell populations, as well as tumour stroma cells.

### 3.8 Protein Signatures Associated With Best Response and Progression‐Free Survival in Osimertinib Treated Lung Cancer Patients

**FIGURE 6:**
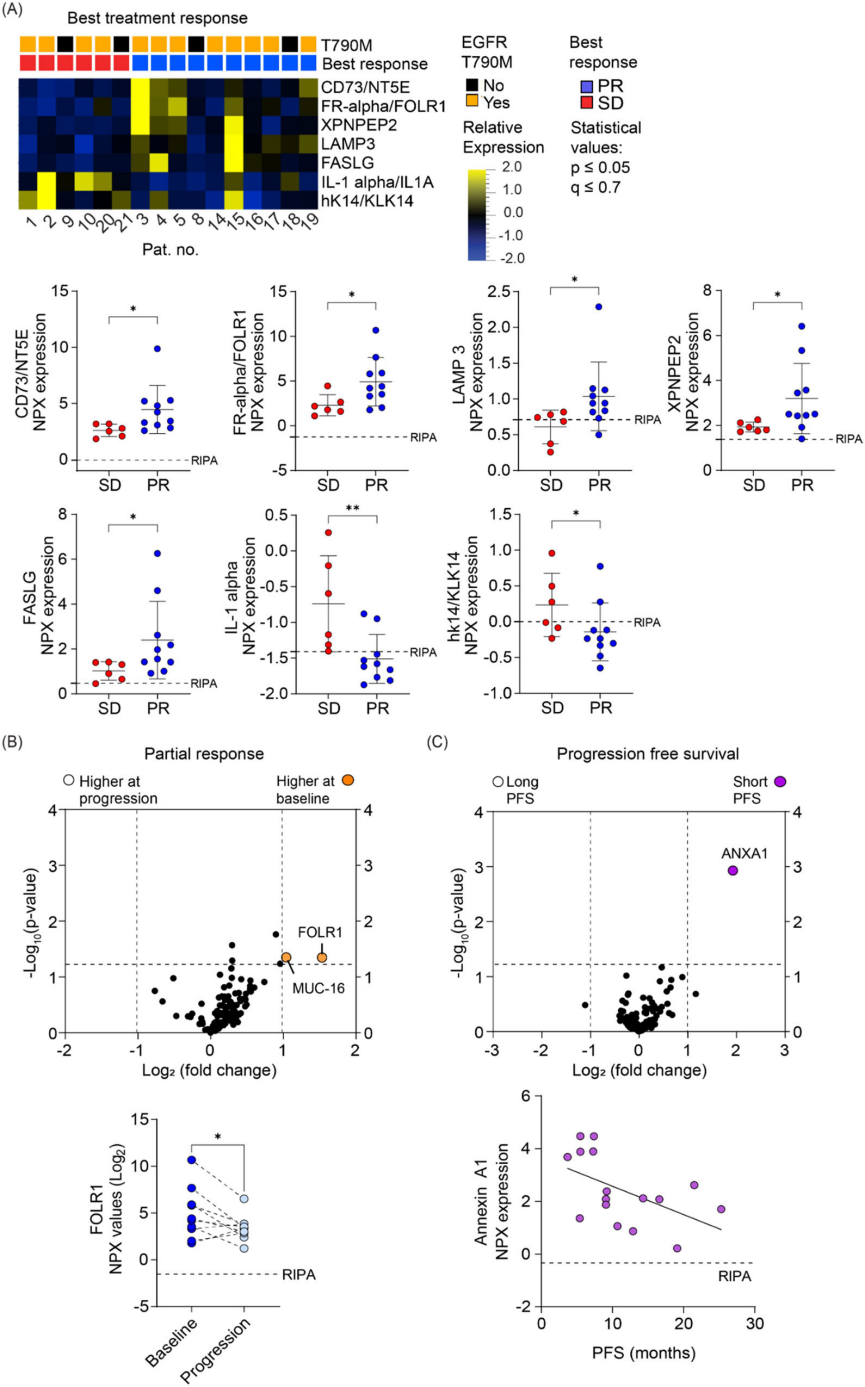
**Extracellular vesicles isolated from serum of non‐small cell lung cancer patients express protein signatures associated with osimertinib treatment**. Extracellular vesicles from NSCLC patient serum were profiled by proximity extension assay (PEA). Proteins were taken into the analyses if expressed over RIPA negative control in 60% of the samples. Five patients were excluded from these analyses; four due to lack of baseline sample (Ptn. #6, #11; #12, #13) and one (Ptn. #7) as this patient progressed without reaching stable disease (SD) or partial response (PR). **(A)** Proteins associated with best treatment response, comparing SD versus PR, using NPX values from the PEA assay are shown. A heatmap of proteins associated with best treatment response using the Qlucore software with Mann–Whitney two‐group *t*‐test statistics (*p* value ≤ 0.05, *q* value ≤ 0.7) is shown (***top panel***). NPX values for individual proteins revealed in relation to best response, grouped as SD or PR. Statistics were calculated using Mann–Whitney two‐group *t*‐test (*; *p* value ≤ 0.05) (***bottom panel***). **(B)** Volcano plots depicting the comparison of NPX values at baseline versus progression in patients with PR (***top panel***). Proteins with significantly higher NPX‐values at baseline as compared to progression were determined by paired two‐group *t*‐test (*p* value ≤ 0.05, fold change > 2). NPX values are indicated for FR‐alpha/FOLR1 and MUC‐16. The expression of FR‐alpha/FOLR1 in serum‐isolated EVs at baseline and progression in individual patients is presented with a paired *t*‐test performed to evaluate the significance (*p* value ≤ 0.05; ***bottom panel***). **(C)** Volcano plot showing proteins associated with long (>8.9 months) or short (<8.9 months) progression‐free survival (PFS) (***top panel***). Linear regression analysis of ANXA1 expression at baseline in relation to PFS (*R* ^2^ = 0.27; *p* value < 0.04; ***bottom panel***).

Next, we aimed to reveal EV protein signatures in the PEA data indicative of osimertinib response or PFS of the patients. First, we focused on proteins related to best response by comparing the EV protein expression levels in patients with stable disease (SD) versus partial response (PR) at baseline. The Mann–Whitney two group analysis (*p* value ≤ 0.05, *q* value ≤ 0.7) revealed seven proteins that associated with best response (Figure 6A), where a higher level of CD73/NT5E, FR‐alpha/FOLR1, X‐prolyl aminopeptidase 2 (XPNPEP2), lysosome‐associated membrane glycoprotein 3 (LAMP3) and fas‐ligand (FASLG) were linked with PR. In contrast, increased interleukin 1 alpha (IL‐1 alpha/IL1A) and kallikrein related peptidase 14 (hk14/KLK14) was associated with SD. These proteins were also significant when analysed individually (Figure 6A **, lower pane**l).

Next, we compared the difference in expression levels between baseline and progression samples in relation to PR (Figure 6B **, upper panel**). FR‐alpha/FOLR1 expression was found to have a lower expression at progression (Figure 6B **, lower panel**), which was not observed in patients with SD (data not shown).

We also analysed EV proteins in relation to PFS by comparing samples from patients with short versus long PFS. The cutoff was set to 8.9 months based on the entire TREM patient cohort (Eide et al. 2020). One protein, ANXA1, was found to have a higher expression in baseline samples from patients with short PFS (*p* value < 0.05, fold change >2, Figure 6C **, upper panel**). Furthermore, linear regression revealed a clear association between PFS in months and the levels of ANXA1 at baseline, with a higher level observed in patients with shorter PFS (*p* value ≤ 0.04; *R* ^2^ = 0.27) (Figure 6C **, lower panel**).

Collectively, our results suggest that protein signatures related to osimertinib response can be revealed in EVs isolated from serum of patients taken prior to osimertinib. However, there is a need to expand the cohort to capture patient heterogeneity and also to use strategies to specifically analyse tumour, immune or stroma cell origin of the EVs to further evaluate these EV proteins as potential biomarkers of osimertinib treatment.

## 4 Discussion

Although the third generation EGFR third generation EGFR tyrosine kinase inhibitor (TKI) osimertinib has improved the outcome for many patients with NSCLC tumours driven by mutated *EGFR* and with advanced disease, emerging treatment resistance is still a major clinical dilemma. Accordingly, non‐invasive treatment monitoring tools are warranted, such as biomarker assessment in patient liquid biopsies. In this work, we aimed to find EVs proteins associated with osimertinib resistance and best treatment response which could constitute putative biomarkers. For that purpose, we first studied the proteome of EVs from a pair of NSCLC cell lines with activating mutations in the *EGFR* gene, alongside the *EGFR T790M* resistance mutation, and with different degrees of osimertinib refractoriness. We furthermore investigated the protein cargo of EVs isolated from serum of patients with mutant *EGFR*‐driven stage IV NSCLC disease when given osimertinib in second‐line.

One of the proteins we found to be associated with osimertinib refractoriness in the *EGFR* mutant cell lines and their released EVs was CSPG4. To our knowledge, this is the first time reporting on CSPG4 in EVs from NSCLC cells, and the role of CSPG4 in context of NSCLC is also limited, as only one report has previously been published in which a TCGA data set was studied, and CSPG4 expression was found to be associated with unfavourable outcome of early‐stage NSCLC (Liu et al. 2024).

Even though, we for the first time, show in this work, siRNA against CSPG4 can reduce cell viability of osimertinib refractory NSCLC cells, CSPG4 has for long been known to regulate cancer hallmarks in other tumour types, such as malignant melanoma (Price et al. 2011, Yang et al. 2019), breast cancer (Wang et al. 2010) and sarcoma (Cattaruzza et al. 2013). Thus, Cattaruzza et al. pointed out phosphorylation of FAK as a downstream result of interactions between CSPG4 and collagen VI, promoting migration and invasion (Cattaruzza et al. 2013) and Yang, et al. showed that a pointmutation in CSPG4 reduced malignant melanoma cell survival and decreased phosphorylation of FAK and ERK (Yang et al. 2019).

Importantly, CSPG4 has also been evaluated as a potential therapeutic target in different tumour types using T‐cell or antibody‐based strategies (Nicolosi et al. 2015, Koopmans et al. 2019, Ilieva et al. 2017, Teppert et al. 2023). For malignant melanoma, a bispecific antibody therapy targeting both PD‐L1 and CSPG4 has been shown to restore the immune visibility of the tumour (Koopmans et al. 2019). In this work, we found that both PD‐L1 and CSPG4 were expressed in and on the EVs isolated from the serum of some of the *EGFR*‐mutated NSCLC patients. Therefore, it would be interesting to evaluate such bi‐specific PD–L1xCSPG4 antibody in the context of osimertinib in mutant *EGFR*‐driven NSCLC.

While CSPG4 was found to be expressed on the serum‐isolated EVs from all patients both at baseline and upon progression, we did not find any association between the expression levels and patient outcome. One could hypothesize that if CSPG4 expression was directly linked to osimertinib responsiveness, higher levels of CSPG4 could be expected in EVs isolated from serum of patients with short PFS and/or OS, but our results did not show such an association. As all the patients were given osimertinib in second‐line following refractoriness to earlier generations of EGFR‐TKIs, it would be possible that an increased CSPG4 expression had already been triggered at as part of a resistance mechanism, possibly masking further changes upon osimertinib treatment refractoriness. Given this, it would be interesting to compare CSPG4 levels in *EGFR* mutant tumours and their released EVs with samples from EGFR‐TKI treatment‐naïve patients at baseline and upon progression. Furthermore, the lack of association between the CSPG4 expression in the EVs and patient outcome could be due to the fact that we analysed the total EVs in the serum, and not only tumour cell derived EVs and hence, the tumour associated changes could be masked, calling for enrichment of tumour derived EVs in further studies.

When analysed by ELISA, the CSPG4 expression on the cell line‐derived EVs exhibited a certain degree of discrepancy compared to the results obtained by the MS analysis. Moreover, when CSPG4 expression was examined by flow cytometry on cells, both H1975 and H1975/OR cells expressed CSPG4 at a similar level, which was in contrast to the MS profiling, where H1975/OR cells were found to have a more prominent expression than H1975 cells. One can hypothesize that the two cell lines exhibit comparable CSPG4 expression levels on their cell surfaces. However, osimertinib‐resistant cells may manifest augmented intracellular expression levels (Feutlinske et al. 2015), which are reflected in their EVs. Further analyses are, however, required to reveal if that is the case.

Mass spectrometry showed that THBS1 and CSPG4 levels co‐vary in H1975 and H1975/OR cells and in their EVs. In relation to RNA‐profiling, our research group has shown that THBS1‐dependent activation of the FAK pathway was implicated in osimertinib resistance, and the GO terms overlapping with CSPG4 and our KEGG pathways (Kosibaty et al. 2022). Given that THBS1 and CSPG4 can converge on FAK signalling, these datasets support a FAK‐centred network contributing to osimertinib refractoriness.

Interestingly, Pietrowska et al. has previously identified THBS1 to be enriched in CSPG4‐captured EVs from plasma of melanoma patients (Pietrowska et al. 2021). Given these findings, it would be interesting to further study the interactome between CSPG4, THBS1, and the FAK‐pathway in the context of osimertinib resistance both *in vitro*, in tumour tissue, and in EVs from serum samples of patients with mutant *EGFR*‐positive NSCLC. Of note, several proteins in the EV protein signature associated with osimertinib resistance in the cell lines have previously been reported to be of importance for cell‐to‐cell or cell‐to‐extracellular matrix (ECM) communication. Among these proteins were perlecan (HSPG2) (Johnson et al. 2024), transgelin (TAGLN) (Liu et al. 2017) and tenascin‐C (TNC) (Schlensog et al. 2023) all which were found to have an increased expression level in the H1975/OR cells and their released EVs. TNC is a glycoprotein that has been demonstrated to promote metastasis in different ways, that is, migration, invasion, proliferation and angiogenesis (Lowy and Oskarsson 2015), and has been implied in the pre‐metastatic niche formation through the activation of macrophages and endothelial cells (Hongu et al. 2022). Interestingly, a higher expression of TNC has been reported in tumour tissue of early‐stage NSCLC cases as compared to adjacent non‐tumour tissue, with a further elevated expression seen in metastatic lesions (Schlensog et al. 2023). Moreover, in vitro studies on *EGFR*‐mutant NSCLC cells support that TNC is a signalling component involved in promoting migration (Schlensog et al. 2023). Taken together, our results suggest that high expression of TNC could be indicative of osimertinib refractoriness.

TAGLN is an intracellular protein that regulates actin and plays a role in cytoskeletal formation, adhesion, migration and proliferation (Jimenez Jimenez et al. 2025). Although TAGLN is reported to be downregulated in some tumour types as part of an early cell transformation process (Sayar et al. 2015), an increased TAGLN expression has conversely been shown to influence proliferation and promote migration and invasion of NSCLC cells (Fu et al. 2020). Our MS analysis of EVs from serum of Ptn. #3 and #5 also revealed these proteins to have a higher expression in Ptn. #3, who had a poor outcome on osimertinib. In conclusion, our results indicate that it may be interesting to further investigate the role of TAGLN, TNC and HSPG2, alongside THBS1 and CSPG4, in the context of osimertinib refractoriness in NSCLC and in particular to expand the MS profiling of EVs to a larger cohort of patients with different outcomes on osimertinib.

We also analysed protein cargo of EVs isolated from serum of patients treated with osimertinib at Karolinska University Hospital as part of the TREM study by PEA profiling. The PEA assay allows analysis of low expressed proteins due to a PCR amplification step (Assarsson et al. 2014, Lundberg et al. 2011) and has been demonstrated to be applicable for analysis of proteins in EVs (Larssen et al. 2017, Indira Chandran et al. 2019). When grouping the patients based on best treatment response (SD vs. PR), several proteins in the baseline samples were found to have a higher expression in EVs from patients with PR, including CD73/NT5E, FR‐alpha/FOLR1, and FASLG.

With respect to FR‐alpha/FOLR1 expression, a decrease was found in EVs from patients with PR at progression versus baseline, while no change was observed in patients with SD. FR‐alpha/FOLR1 is a protein that binds folate, needed for DNA and RNA synthesis and cell proliferation (Locasale 2013). Consequently, a higher amount of folate is needed in rapidly dividing cells, such as tumour cells (Gonzalez et al. 2024). The folate and FR‐alpha/FOLR1 signalling axis have in different cancer cells been shown to activate multiple proliferative controlling kinases, for example, JAK/STAT, SRC and ERK (Nawaz and Kipreos 2022). Interestingly, FR‐alpha/FOLR1 has previously been reported to have a higher expression in LUAD cases with mutant *EGFR* (Iwakiri et al. 2008, Tamura et al. 2020, Nunez et al. 2012).

Under the assumption that the protein expression in the EVs is reflective of tumour‐derived expression patterns, a decreased FR‐alpha/FOLR1 level in EVs at progression could indicate a more effective treatment regimen, as the proliferation rate slows down in conjunction to the decreased cellular influx of folate. Thus, it makes sense for patients with better response status to experience a decrease in FR‐alpha/FOLR1 expression over time.

We also found a higher CD73/NT5E expression in the EVs isolated from serum of the patients that showed PR after osimertinib treatment. Moreover, CD73/NT5E expression in the EVs also correlated with EVs levels of PD‐L1. CD73/NT5E is recognized for its immune suppressive role in the TME acting in part via regulation of adenosine levels (Saigí et al. 2023). Interestingly, CD73/NT5E expressed on EVs isolated from plasma of malignant melanoma patients, was indeed reported to participate in the production of adenosine, thereby influencing the TME (Turiello et al. 2022, Hong et al. 2025).

In our cohort, CD73/NT5E expression was detected in EVs from all of the patient serum samples and we found that patients obtaining PR had a higher expression of CD73/NT5E in their EVs at baseline compared to patients who reached SD. CD73/NT5E has previously been reported in LUAD tumours and was demonstrated to have a higher expression in *EGFR‐*mutant tumours versus those with wildtype *EGFR*, with a further increase seen upon EGFR‐TKI refractoriness (Ferrara et al. 2022, Kim et al. 2023, Le et al. 2021). Moreover, CD73/NT5E has also been reported in NSCLC cells to be involved in signalling cascades which control proteins involved in EGFR‐TKI resistance including AXL (Zhu et al. 2024) and MET (Yoshida et al. 2022).

Preclinical results show that osimertinib regulates CD73/NT5E (Tu et al. 2022), and an effect of a bispecific CD73xEGFR antibody in vitro and in mice (Ploeg et al. 2023). These results together with clinical programs testing anti‐CD73 antibody oleclumab treatment in NSCLC, alone or combined with immune checkpoint inhibitors (Bendell et al. 2023, Barlesi et al. 2024, Besse et al. 2024), suggests that an interesting way to continue would be to study these treatments together with osimertinib in *EGFR T790M* positive NSCLC.

In our study, we analysed total EV content in the serum, including both tumour and immune cell derived EVs and found that a high CD73/NT5E expression on EVs was linked to PR. Albeit we tried to selectively study tumour‐derived EVs using either capturing with CSPG4‐antibody conjugated beads or by analysing the presence of *EGFR T790M* mutation in RNA in the EVs, we did not manage those verifications at this point. Yet this is of high relevance as it would strengthen the putative EVs biomarker directly to tumour cells. Moreover, as CD75/NT5E expression on EVs was reported to regulate the immune cells and thereby change their anti‐tumour activity (Turiello et al. 2022, Hong et al. 2025), and given a recent report showing a strategy to target EV‐carried CD73 effects in other tumour types using a bispecific antibody toward CD73xEpCAM (Ploeg et al. 2021), it would also be interesting to evaluate this effect in the context of osimertinib resistance.

We also found that a high EVs expression of ANXA1 was associated with short PFS. ANXA1 has previously been correlated to poor survival in NSCLC of different histological subtypes including LUAD (Biaoxue et al. 2012). The protein has also been explored in urinary bladder/urothelial carcinoma (UC), where it was found to be linked to high EGFR expression (Li et al. 2022). Interestingly, the same study explored ANXA1 mechanistically in UC cell lines and showed that shRNA‐mediated ablation resulted in decreased cell survival, impaired migration and invasion capacity, and decreased phosphorylation of EGFR was evident (Li et al. 2022). Moreover, ANXA1 has been suggested to be a therapeutic target of different solid tumours, including LC. One example is a monoclonal antibody MDX‐124, which currently is evaluated in clinical phase Ib trial (ISRCTN78740398). Given our EVs results of ANXA1, it would be of interest to analyse this protein in a larger cohort of *EGFR*‐mutant NSCLC patients undergoing osimertinib treatment.

We revealed PD‐L1 expression in serum‐isolated EVs before osimertinib and also upon relapse. However, only a fraction of the analysed samples had an expression level over RIPA negative control and the EV expression levels were overall rather low. This is not surprising as it has been shown that NSCLC patients with activating *EGFR* mutations express less PD‐L1 than patients with an *EGFR* wildtype phenotype (Le et al. 2021). Among the proteins associated with PD‐L1 expression in the EVs at baseline were FR‐alpha/FOLR1 as well as EPHA2, the latter which has been linked to refractoriness to first generation EGFR‐TKIs in NSCLC model systems (Amato et al. 2016). Expanding the analysis of these PD‐L1 associated proteins in EVs and their potential signalling interconnections in context of the immune system using a larger patient cohort, could be ways forward to evaluate their biomarkers potential. Another way could be to analyse how these proteins change after the first treatment cycle of osimertinib. As we did not only study tumour derived EVs, the proteins that we found to be associated with PD‐L1 or CD73/NT5E expression or to best treatment response could also be of immune cell origin. Using the Human Protein Atlas (https://www.proteinatlas.org/), we found that 17 out of the 22 identified proteins were expressed in different immune cells while FR‐alpha/FOLR1, Cyr61/CCN1, CEACAM5, KLK14 and IL‐1 lacked such expression indicating that their origin may be tumour cells or related to tumour stroma. To further understand the different expression levels in the context of cell origin, one would in future studies need to do assessment of the EVs using capturing or staining for markers that are specific for such cell subsets.

It should be noted that there are a number of limitations of our study, such as the small patient cohort size with large heterogeneity in terms of treatment prior to the given osimertinib regimen and the diverse metastatic burden of the patients. Hence, the proteins revealed in our signatures need to be further studied and evaluated in an expanded patient cohort of serum‐isolated EVs. Moreover, to be able to better assess the effect of osimertinib treatment response on the EV proteome, the patients included in such analyses should preferably have been given a more similar treatment regimen prior to sample collection, and/or share a more homogenous metastatic burden. Moreover, as tumour derived EVs just constitute a minor proportion of the total EV population found in plasma or serum, applying selective isolation strategies based on tumour‐, immune‐, or stroma cell–specific markers for capturing and thereafter analysing the EV protein cargo in context patient outcome on osimertinib could clearly be one way ahead.

## 5 Conclusion

In summary, our analysis of protein cargo from EVs isolated from mutant *EGFR*‐driven NSCLC cells revealed the presence of a protein signature related to osimertinib resistance, including CSPG4, HSPG2, TAGLN, THBS1 and TNC and indicated involvement of biological pathways such as the focal adhesion and proteoglycans in cancer pathways. CSPG4 expression was also evident in EVs from NSCLC patient serum samples but showed no association to patient outcome in this heterogeneous patient cohort. Moreover, by PEA profiling of the EV cargo, we were able to identify protein signatures linked to the best response to osimertinib, among them CD73/NT5E and FR‐alpha/FOLR1. We also found increased ANXA1 EVs levels to be associated with short PFS. In summary, our EVs protein cargo profiling has revealed multiple proteins, including ANXA1, CD73/NT5E, CSPG4, FR‐alpha/FOLR1, HSPG2, THBS1 and TNC to be differentially expressed in relation to osimertinib refractoriness.

## Author Contributions

Conceptualization: A. C. V., P. Hå., R. L. and K. V. Supervision: A. C. V., P. Hå., A. D., R. L. and K. V. Methodology: A. C. V., P. Hå., S. J., P. Hy., B. F., A. V., N. A., S. S. S., F. S. and A. D. Validation: A. C. V., P. Hå. S. J., P. Hy. and N. A. Formal analysis: A. C. V., P. Hå., S. J., P. Hy., N. A., B. F., A. V., I. J. Z. E, S. S. S., F. S., and A. D. Patient recruitment and patient data analysis: I. J. Z. E., L. D. P., S. E., O. T. B. Resources/Funding: PHy., A. D., L. D. P., S. E., O. T. B., R. L. and K. V. Writing – original draft preparation: A. C. V., P. Hå., S. J., and K. V. Review: Hy., B. F., A. V., I. J. Z. E., N. A., S. S. S., F. S., A. D., L. D. P., S. E., O. T. B. and R. L. All authors agree to the submitted version of the study.

## Conflicts of Interest

O.T.B have received free drug/trial support for the study per institution from AstraZeneca including consultancy/lecture/presentation/data safety monitoring or advisory board. I.J.Z.E. has received lecture honoraria from Pfizer and Bristol Meyer Squibbs and for advisory board appointments from AstraZeneca and Janssen. The other authors have no conflict of interest in relation to this work.

## Supporting information




**Supplementary Material**: jev270219‐sup‐0001‐SuppMat.docx

## Data Availability

The MS‐or PEA raw data corresponding to the data shown in this work can be obtained from the corresponding authors upon request.
