# Supplementary material for: Profiling of Extracellular Vesicles of Non‐Small Cell Lung Cancer Reveals Proteins Associated With Osimertinib Resistance
Source: J Extracell Vesicles. 2026 Jan 25;15(1):e70219. doi: 10.1002/jev2.70219 (PMC12832073; doi:10.1002/jev2.70219)
Supplement: Supplementary file 1 — Supplementary Material: jev270219‐sup‐0001‐SuppMat.docx [file JEV2-15-e70219-s001.docx]

**Profiling of extracellular vesicles of non-small cell lung cancer reveals proteins associated with osimertinib resistance**

Albano Cáceres-Verschae^1^*^#^, Petra Hååg^1^*, Sofia Joelsson^1^, Per Hydbring^1^, Bo Franzén^1^, Ákos Végvári^2^, Inger Johanne Z. Eide^3,4^, Nupur Agarwal^1^, Siddharth Sourabh Sahu^5^, Fredrik Stridfeldt^5^, Luigi De Petris^1,6^, Apurba Dev^7^, Simon Ekman^1,6^, Odd Terje Brustugun^3,4^, Rolf Lewensohn^1,6^, and Kristina Viktorsson^1#^.

^1^ Dept of Oncology and Pathology, Karolinska Institutet, Stockholm, Sweden.

^2^ Dept of Medical Biochemistry and Biophysics, Karolinska Institutet, Stockholm, Sweden.

^3^ Section of Oncology, Vestre Viken Hospital Trust, Drammen, Norway.

^4^ Dept of Cancer Genetics, Institute for Cancer Research, Norwegian Radium Hospital, Oslo University Hospital; Dept of Clinical Medicine, University of Oslo, Oslo, Norway.

^5^ Department of Applied Physics, School of Engineering Sciences, KTH Royal Institute of Technology, Stockholm, Sweden.

^6^ Theme Cancer, Patient area Head, Neck, Lung and Skin Cancer, Karolinska University Hospital, Stockholm, Sweden.

^7^ Division of Solid-State Electronics, Department of Electrical Engineering, Uppsala University, 75121 Uppsala, Sweden.

* These two authors contributed equally to this work.

**# Corresponding authors**:

Dr Albano Cáceres-Verschae; albano.caceres.verschae@ki.se or

Dr Kristina Viktorsson; kristina.viktorsson@ki.se

Department of Oncology/Pathology, Theme Cancer, J6:20

Karolinska Institutet

Visionsgatan 4,

S-171 64, Solna, Sweden

**Supplementary material and methods:**

***Assessment of osimertinib effect on cell viability***. The concentration of osimertinib which inhibited cell viability by 50% (Inhibitory concentration 50 (IC50)) at 48 h were assessed in H1975 and in H1975/OR cells. In brief, in a 96-well plate 5 x10^3^ cells were seeded per well and different concentrations of osimertinib were added. As a negative control 0.01% v/v DMSO was used (Sigma-Aldrich, Burlington, Massachusetts, US, cat. #D2438). After fixing the cells by trichloroacetic acid solution 40% w/w for 1 h and washing in tap water three times, sulforhodamine B solution (SRB; Sigma-Aldrich, cat. #230162) was added followed by 1 h incubation. Acetic acid diluted to 1% (Sigma-Aldrich, cat. #A6283) was applied and thereafter the plate was left overnight to dry. The dye was resuspended in 100 nM Tris-Base and the resulting absorbance monitored at 456 nm (SpectraMax^®^ I3, Molecular Devices, Silicon Valley, CA, United States). The effect of osimertinib was calculated relative to negative DMSO control. For cell harvesting at the time of EVs isolation, photos were taken at final magnification of 40x, and cells were counted with trypan blue to determine cell viability.

***Mass spectrometry profiling of extracellular vesicle protein cargo***. For the liquid chromatography-tandem mass spectrometry (LC-MS/MS) profiling of extracellular vesicle (EV) protein cargo, EVs from three biological replicates of H1975 or H1975/OR cells were solubilized in 100 µL of 8 M urea (Sigma-Aldrich, cat. #51457) in 50 mM Tris, pH 8.5 supplemented with 1 µL of 100x protease- and phosphatase inhibitors (Pierce, cat. #78440). Samples were sonicated with 2% Protease MAX (Promega, Nacka, Sweden, cat. #V2071) added in 20% acetonitrile (ACN, Sigma-Aldrich, cat. #34998) and 50 mM Tris-HCl buffer for 10 min. After clearing the lysate by centrifugation, bicinchoninic acid assay (BCA) assay was used to assess the protein concentration (Thermo Fisher Scientific, cat. #23225). Samples were prepared by taking 10 µg protein from each biological replicate, reducing for 45 min at 37 °C in 2.5 µL of a 250 mM dithiothreitol solution (Sigma-Aldrich, cat. #D9779) and alkylating for 30 min in dark in 3 µL of 0.5 M iodoacetamide (Sigma-Aldrich, cat. #I6125). Proteolytic digestion of the EV lysates started with addition of 3 µg of 0.1 µg/µL endoproteinase Lys-C (Wako, Japan, cat. #125-05061) and incubating at 24 °C during a 5 h period after which 215 µL of 50 mM Tris-HCl buffer was added. The digestion was completed using 5 µL of 0.1 µg/µL sequencing grade trypsin (Promega, cat. #V5113) per sample followed by a 16 h incubation at 37 °C after which 15 µL of formic acid (FA, Sigma-Aldrich, cat. #5.33002) was added to terminate digestion. Desalting was carried out on a C18 HyperSep plate (Thermo Fisher Scientific, cat. #60300-425), followed by drying the samples in a Vacufuge vacuum concentrator (Eppendorf, Hamburg, Germany).

For the LC-MS/MS analysis an Ultimate 3000 nanoUPLC system was linked with a Q Exactive™ HF hybrid quadrupole-Orbitrap mass spectrometer (Thermo Fisher Scientific). Proteins from the EV samples (3.5 µg of total protein) digested into peptides were subsequently added for separation on a 50 cm long EASY-Spray C18 column applying a linear gradient from 4% solvent B (98% ACN/0.1% FA) in 90 min. The mass spectrometer was set up as previously described [1], except for the mass range which was 375 to 1700 *m/z* targeting 1 x10^6^ ions with an 80 ms maximum injection time. Fragmentation of precursor ions in the higher-energy collisional dissociation (HCD) was achieved at 28% collecting fragment ions at a mass resolution of 30,000.

Analysis of the MS raw data was carried out with Proteome Discoverer v3.0 (Thermo Fisher Scientific) with a MS/MS search on Amanda 2.0 search engine against a human consensus protein database (SwissProt, v2023-02-09). The identification of the proteins was based on peptide sequences allowing full tryptic digestion in which two missed cleavage sites were set as limitation, with mass tolerance to 10 ppm for precursor ions and 0.02 Da fragment ions. Methionine oxidation and asparagine/glutamine deamidation was applied as dynamic modifications while cysteine carbamidomethylation was set to static. To calculate the false discovery rate (FDR) the Percolator node in Proteome Discoverer was applied and data was filtered with a 5 % cutoff. For proteins to be taken into the further analyses they had to be identified by at least two peptides. Label-free quantification of proteins was achieved by aligning acquired runs using match between run function and calculating protein abundances based on the precursor peptide intensities. The obtained raw protein abundances were used for further comparisons. The serum-isolated EVs of two patients (Ptn. #3 and Ptn. #5), at baseline and progression, were analyzed in a similar way but starting with an equal number of EVs (1.2 x10^9^) and using 2.5 µg protein/sample for the C18 column step and further on. Here proteins were also taken into the analysis only when revealed by two peptides and expressed in the two or more of the four analyzed samples.

***Mass spectrometry protein profiling of cell extracts****.* The MS-based protein profiling procedure of the cell lysates from H1975 and H1975/OR prior or post osimertinib treatment was prepared essentially as previously reported [1]. For annotation of vendors for the reagents, please see above. In brief, cell pellets were lysed with 106-318 µL of 7.5 M urea in 100 mM Tris-HCl (pH 8.5), 0.1% ProteaseMAX with 100x protease and phosphatase inhibitors with volume depending on the pellet size. After sonication for 10 min in water bath VibraCell probe was used for sonicating the samples at 20% amplitude with 2 s on-off pulse for 40 s. The samples were spun down (12,000 *g* at 4°C for 10 min) and BCA assay used to determine the protein concentration. Alkylation was carried out on an aliquot of 25 µg of each sample in a volume of 3 µL of 250 mM dithiothreitol and first incubating at 37°C for 45 min followed by addition of 4 µL of 0.5 M iodoacetamide and a further incubation for 30 min at room temperature in the dark. For proteolytic digestion, 5 µL of 0.1 µg/µL sequencing grade trypsin were used and the samples incubated overnight at 37°C while rotating. FA at final concentration of 5% was used to terminate the digestion followed by cleaning the samples on a C18 HyperSep plate and drying them up by a Vacufuge vacuum concentrator.

For the tandem mass tag (TMT) labeling the dried samples were solubilized in 70 µL of 50 mM triethylammonium-bicarbonate, pH 8.0, TMTpro reagents (100 µg, Thermo Scientific, dissolved in 30 µL of anhydrous ACN) were added to the samples in scrambled order and incubated at room temperature for 2 h while rotating. To terminate the labeling hydroxylamine (11 µL, final concentration 0.5 %) was added to the samples during a 15 min incubation. One analytical sample was thereafter made by combining the individual samples and drying them down using a vacuum concentrator, followed by cleaning as described above. The sample, containing digests of all the biological replicates, was subsequently fractionated by high-pH reversed-phase chromatography. The combined TMTpro-labeled sample was dissolved in 50 µL of 20 mM ammonium hydroxide, and loaded onto a Acquity bridged ethyl hybrid C18 HPLC column (Waters, Solna, Sweden). For separation a linear gradient consisting of 5–60% of 20 mM ammonium hydroxide in ACN (pH 9.0) over a time span of 48 min and with a flow rate of 200 µL/min was used. A UV detector at 214 nm was employed to monitor the chromatographic performance. Collection of fractions was made every 30 s into a 96-well plate. This was followed by combining the fractions into 12 analytical samples by concatenating the eight fractions, which represented the peak peptide elutions. Each peptide fraction was dissolved in solvent A (2 % ACN, 0.1 % FA in water) and 2 µg was injected onto a 50 cm EASY-Spray C18 column, in which the peptides were separated by a gradient of 4–26 % solvent B at a flow rate of 300 nL/min over 90 min. A Q Exactive HF hybrid quadrupole-Orbitrap mass spectrometer was used to get the mass spectra of the peptides by scanning *m/z* 350–1800 at R=120,000 (at *m/z* 200), which targeted 5x10⁶ ions with a 100 ms max injection time. HCD fragmentations were carried out on the top 17 precursor ions (charge 2+ to 7+) using a dynamic exclusion of 45 s. Tandem mass spectra were acquired at R = 60,000, targeting 5x10⁵ ions with a 54 ms allowed injection time, 1.4 Th quadrupole isolation width, and 34% normalized collision energy. The collected raw data were analyzed by Proteome Discoverer v3.0 using the MS Amanda 2.0 search engine towards the human protein database (SwissProt, v2023-02-09). For full tryptic digestion the allowed missed cleavage sites were set to two and the precursor and the fragment ion mass tolerance were limited 10 ppm and 0.02 Da, respectively. As fixed modification carbamidomethylation of cysteine was set while the dynamic alterations were TMTpro on lysine and N-termini, deamidation of asparagine and glutamine as well as methionine oxidation. The results were searched in Proteome Discoverer initially using a filtration of 5% FDR by application of the Percolator node. For identification of a protein in the sample at least two peptides needed to be detected while the protein abundances were quantified by making use of the TMT-reporter ion abundances of the peptides.

***Methods of protein data normalization***. The TMTpro-labeled mass spectrometry was processed and normalized before further analysing the data.

The normalization of the TMT-labeled mass spectrometry raw protein abundance data of the H1975 or H1975/OR cell extracts as presented in **Figure 3** was done as shown in (**S1**).

$A_{Normalized}= \frac{A_{n,x}}{M_{n}}* M_{tot}(S1)$

A_Normalized_ = Normalized protein abundance

A_n,x_ = The raw protein abundance for protein x in sample n

M_n_ = Median value of all raw protein abundance values in sample n

M_tot_ = Median value of all raw protein abundance values in all samples

**Reference**

1. Shafi AM, Végvári Á, Zubarev RA, Penha-Gonçalves C. Brain endothelial cells exposure to malaria parasites links type I interferon signalling to antigen presentation, immunoproteasome activation, endothelium disruption, and cellular metabolism. Front Immunol. 2023 Mar 13;14:1149107. doi: 10.3389/fimmu.2023.1149107. Erratum in: Front Immunol. 2023 Nov 28;14:1331366. doi: 10.3389/fimmu.2023.1331366.

2. Eide, I. J. Z., Å. S. Helland, S. Ekman, et al. 2020. “Osimertinib in T790M‐Positive and ‐Negative Patients With EGFR‐Mutated Advanced Non‐Small Cell Lung Cancer (The TREM‐Study).” *Lung Cancer* 143: 27–35.

**Supplementary tables and figures**

**Table S1 - Protein abundance data of extracellular vesicles isolated from cell culture media of non-small lung cancer H1975 and H1975/OR cells**. Raw protein abundance obtained by mass spectrometry of the protein identities found in extracellular vesicles isolated from H1975 and H1975/OR cell culture media prior- and post osimertinib as presented in **Figure 1-2** are given.

**
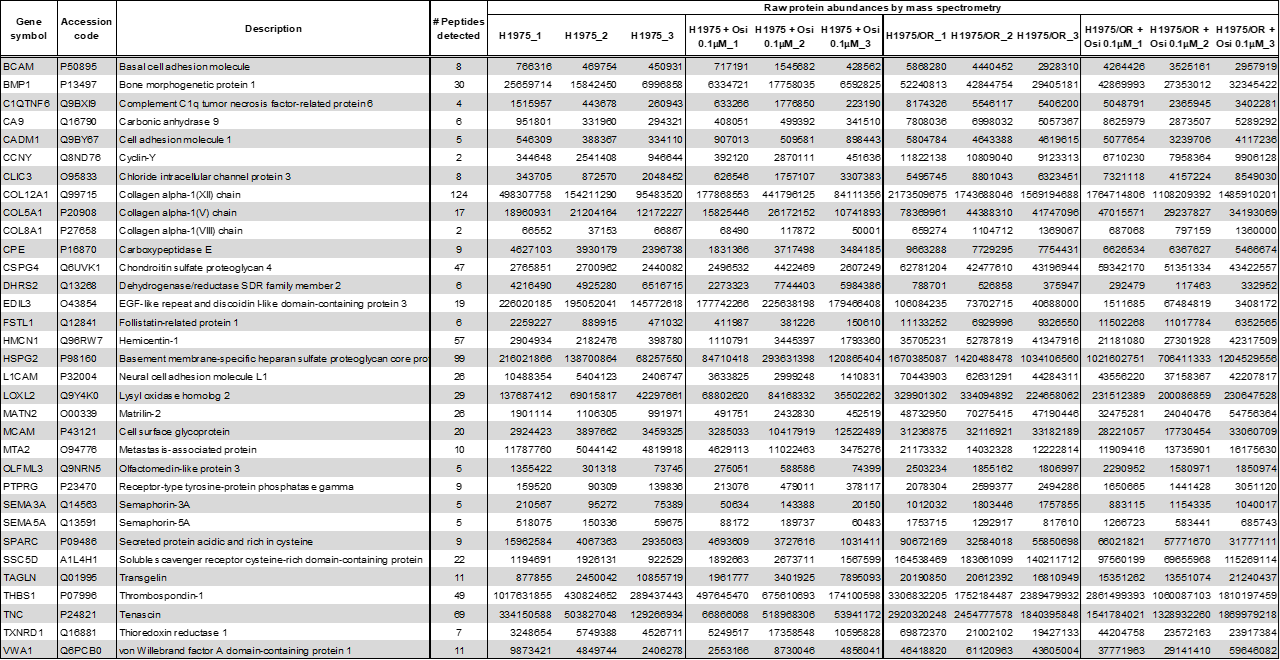
**

**Table S2 - Machine learning algorithms applied for validation of osimertinib associated protein signatures in extracellular vesicles in cell lines**. The artificial intelligence (AI) based classifiers K-nearest neighbor, Random Tree, Support Vector Machines and Extreme Gradient Boosting were applied on the mass spectrometry data of the extracellular vesicles isolated from cell culture media of the H1975 and H1975/OR prior and post osimertinib for *in silico* validation of identified proteins in the signature (Figure 1E). The signature proteins from analysis are presented in “Variables used by classifier” section. All of the proteins revealed were related to H1975/OR independently of osimertinib treatment.


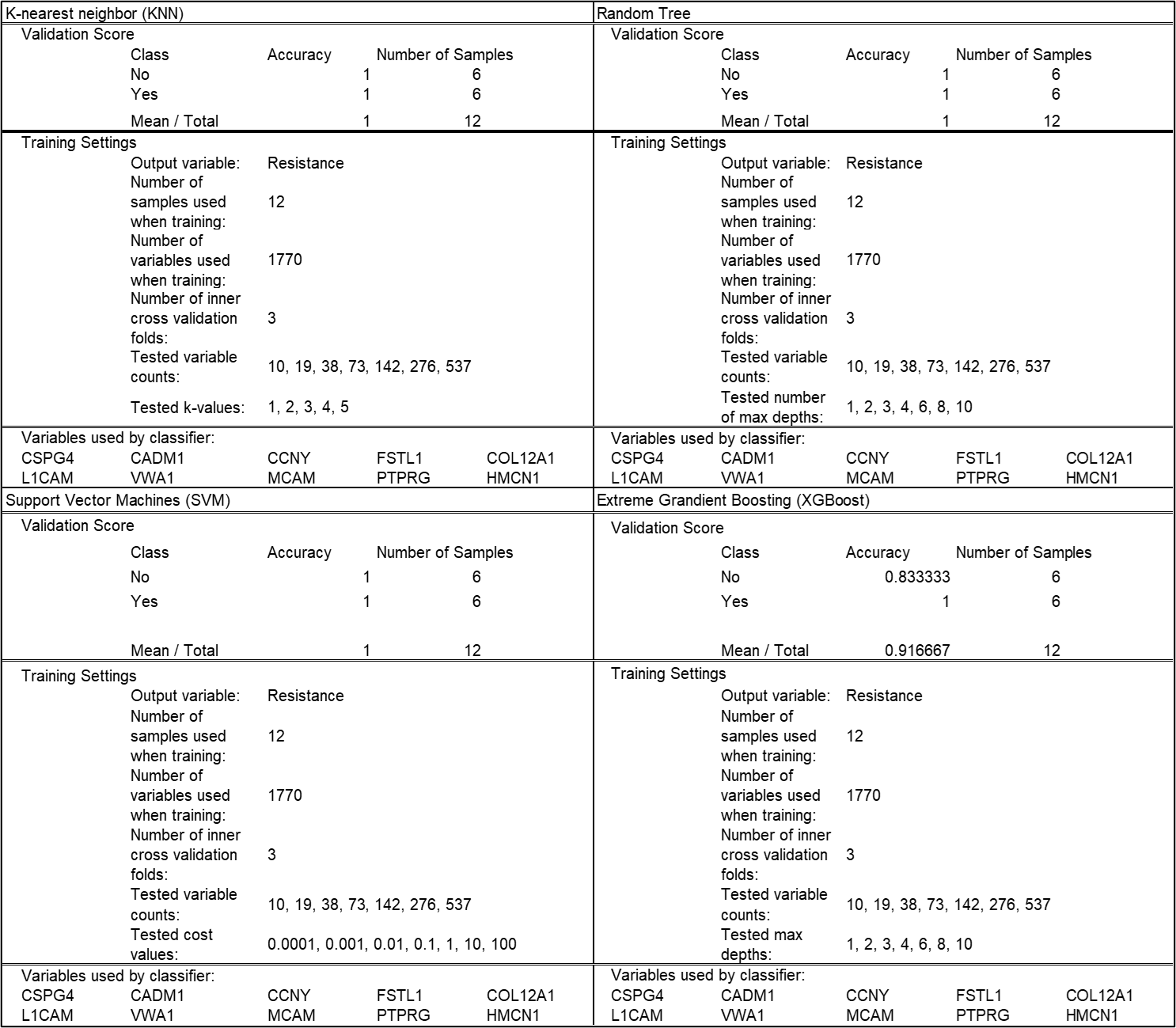


**Table S3 - Protein expression in extracellular vesicles isolated from serum of non-small cell lung cancer patients with different outcomes**. Proteins with higher expression (Log_2_(FC) = ≤-1 or 1≤) in extracellular vesicles (EVs) isolated from serum of two non-small cell lung cancer (NSCLC) Ptn. #3 with Progression Free Survival (PFS): 5.5 months; Overall survival (OS): 9.4 months, and Ptn. #5 with PFS: 16.5 months; OS: 37.1 months at baseline were analyzed by mass spectrometry (MS). Protein identities related to osimertinib resistance in EVs isolated from cell culture media of H1975/OR cells shown in **Figure 1** and **Figure 2** are marked in red.


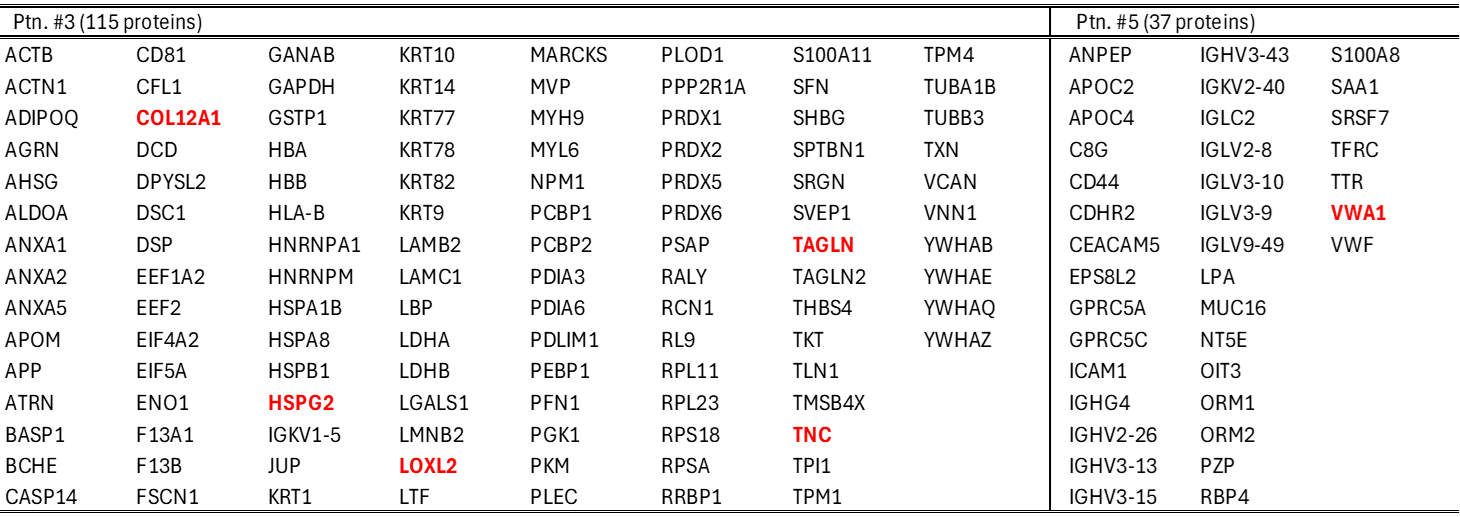


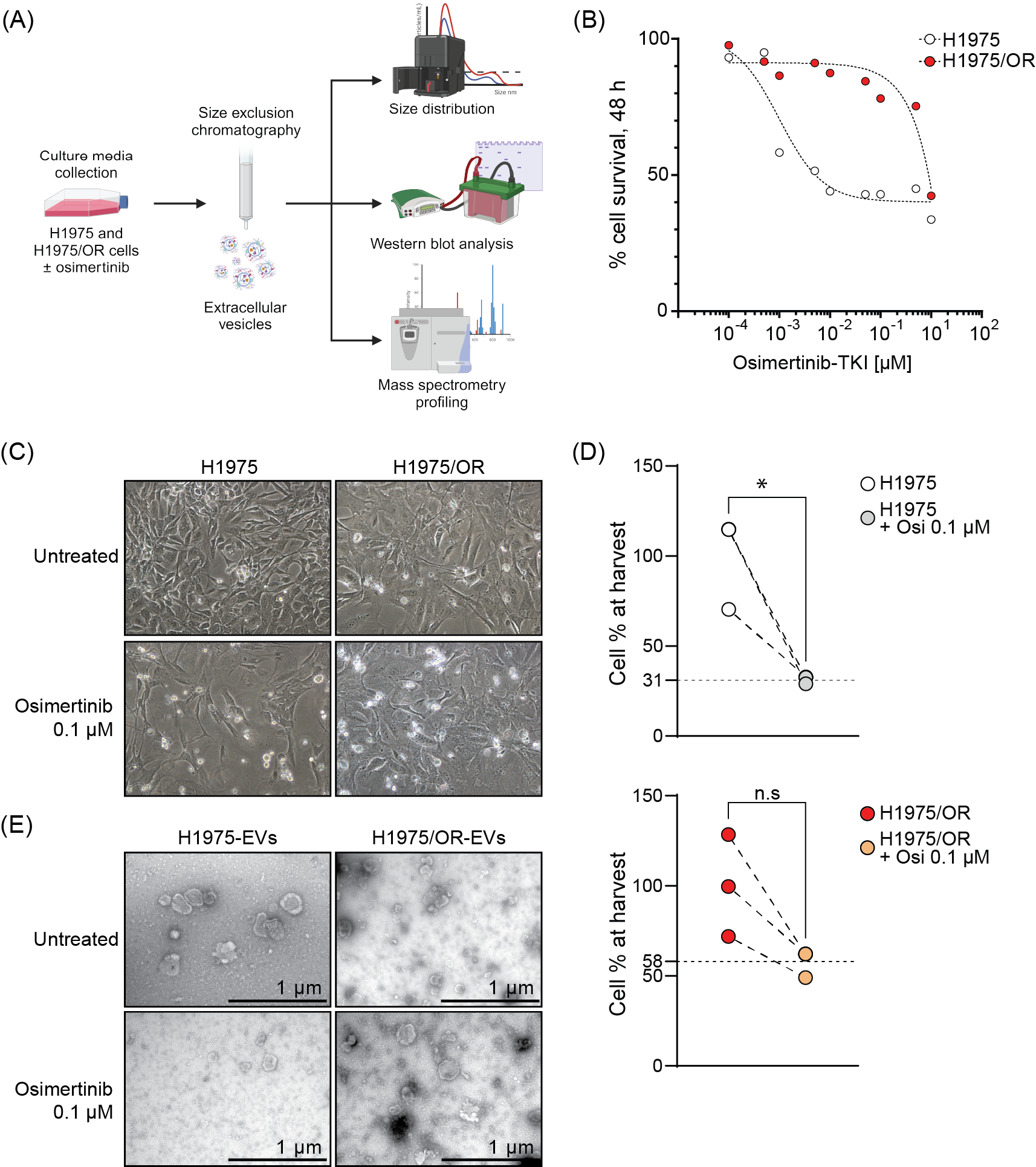


**Figure S1 - Study outline and osimertinib response in H1975- and H1975/OR cell lines**. **(A)** Extracellular vesicles (EVs) from H1975 or H1975/OR cell culture media prior and post osimertinib treatment were isolated by size exclusion chromatography (SEC). Nanoparticle tracking analysis (NTA) was applied to assess size (nm) and amount (particles/mL). EVs proteins were analyzed by western blot and profiled by mass spectrometry followed by bioinformatic processing and validation. **(B)** Cell survival after exposure to indicated osimertinib concentrations for 48 h was monitored by Sulforhodamine B Assay. Data shown are the mean of four measurements, two biological experiments, each with two technical replicates. Inhibitor concentration vs. response is indicated as % of cell survival. The concentrations that inhibited cell survival by 50% (% survival at 48 h) relative to DMSO control were 0.1 µM for H1975 cells and 8.6 µM for H1975/OR cells, respectively. **(C)** Representative images of cells at the time of harvest at 48 h post osimertinib addition. Images were captured at 40x magnification. **(D)** Graph presenting the % viable cells at harvest after average normalization of untreated cells set to 100% for untreated or osimertinib treated H1975 or H1975/OR cell. Three biological replicates are shown, paired t-test, ∗ p-value < 0.05. **(E)** The size and morphology of the isolated particles were studied by transmission electron microscopy (TEM). Images show particles isolated from cell culture media of H1975 and H1975/OR cells treated with or without osimertinib . Sizes are indicated by the scale bar of 1 µm.

**
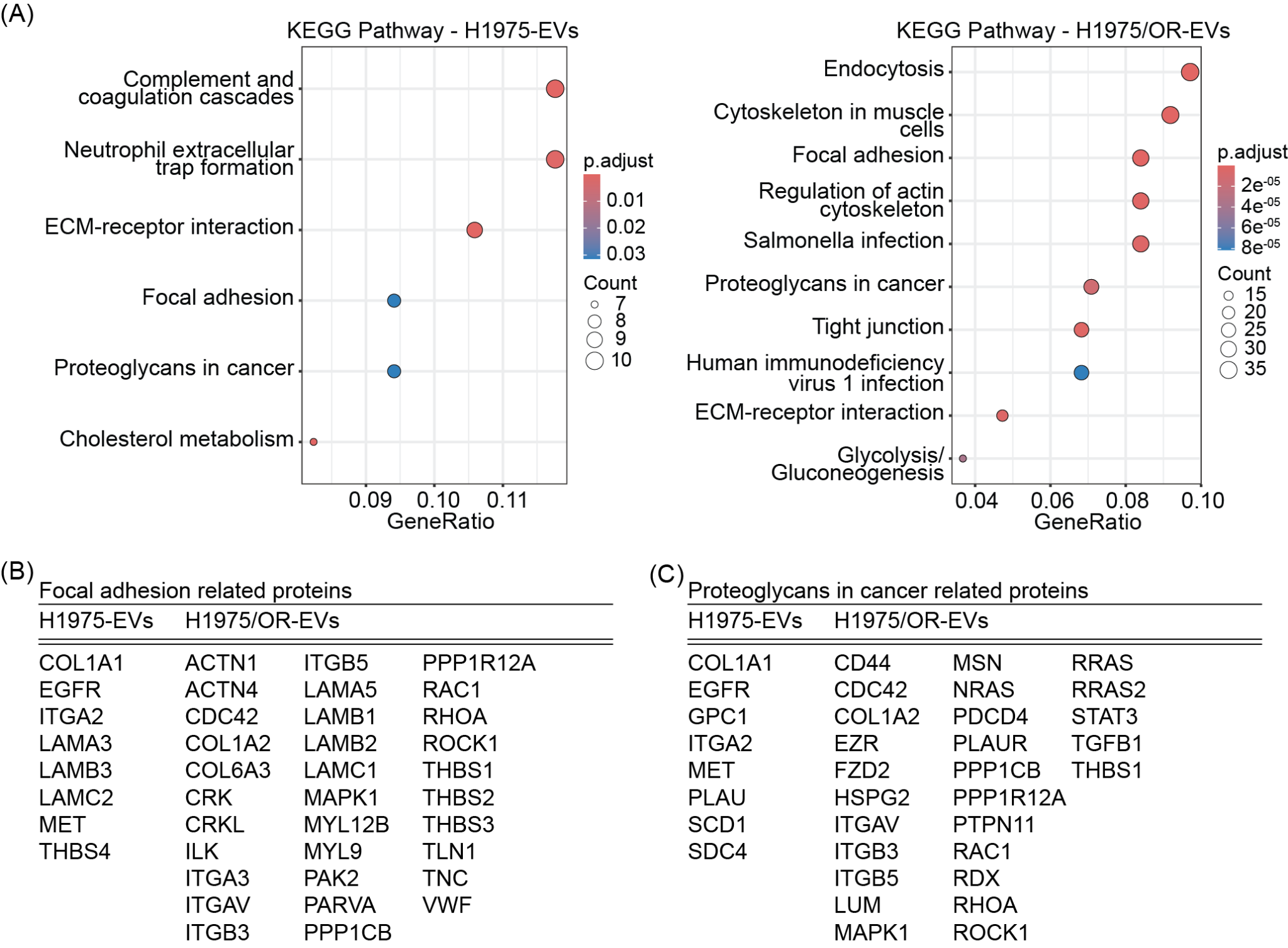
**

**Figure S2 - KEGG pathways of extracellular vesicles from non-small cell lung cancer cell lines**. **(A)** The top 10 ranked pathways in the KEGG enrichment analysis of proteins that had a higher expression (Log_2_(FC) = ≤-1 or 1≤) and -Log_10_(0.05) in extracellular vesicles (EVs) from either cell line (H1975-EVs vs. H1975/OR-EVs) are shown. List of proteins linked to **(B)** focal adhesion signaling and **(C)** proteoglycans in cancer pathways.

**
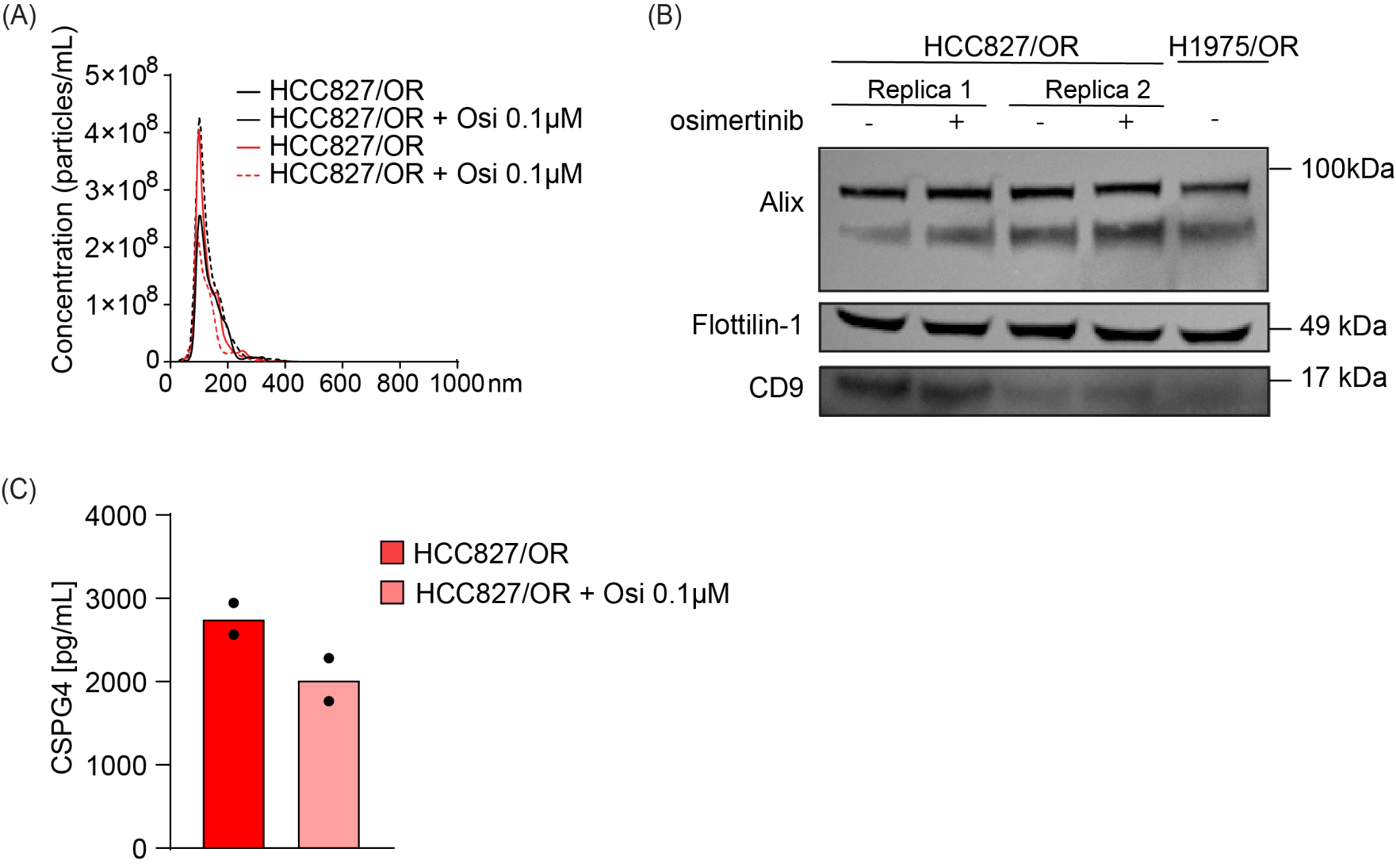
**

**Figure S3 - Extracellular vesicles isolated from mutant EGFR non-small cell lung cancer cell line HCC827 express chondroitin sulfate proteoglycan 4**. HCC827/OR cells were either treated with osimertinib (0.1 µM, 48 h) or not and the cell culture media collected for isolating their released extracellular vesicles (EVs). Data from two biological replicates are shown. **(A)** The size of the particles (in nm) and their concentration (particles/mL) as analyzed by Nanoparticle Tracking analysis are presented. **(B)** Expression of alix, flotillin-1 and CD9 in EVs were confirmed by western blot. 4 x10^8^ EVs were analyzed with H1975/OR EVs as control. **(C)** Protein quantification of CSPG4 by ELISA on 1 x10^9^ non-lysed EVs from untreated HCC827/OR cells (2755 ± 380 pg/mL) or after 48 h osimertinib treatment (2023 ± 515 pg/mL). Data is based on two biological replicates.

**
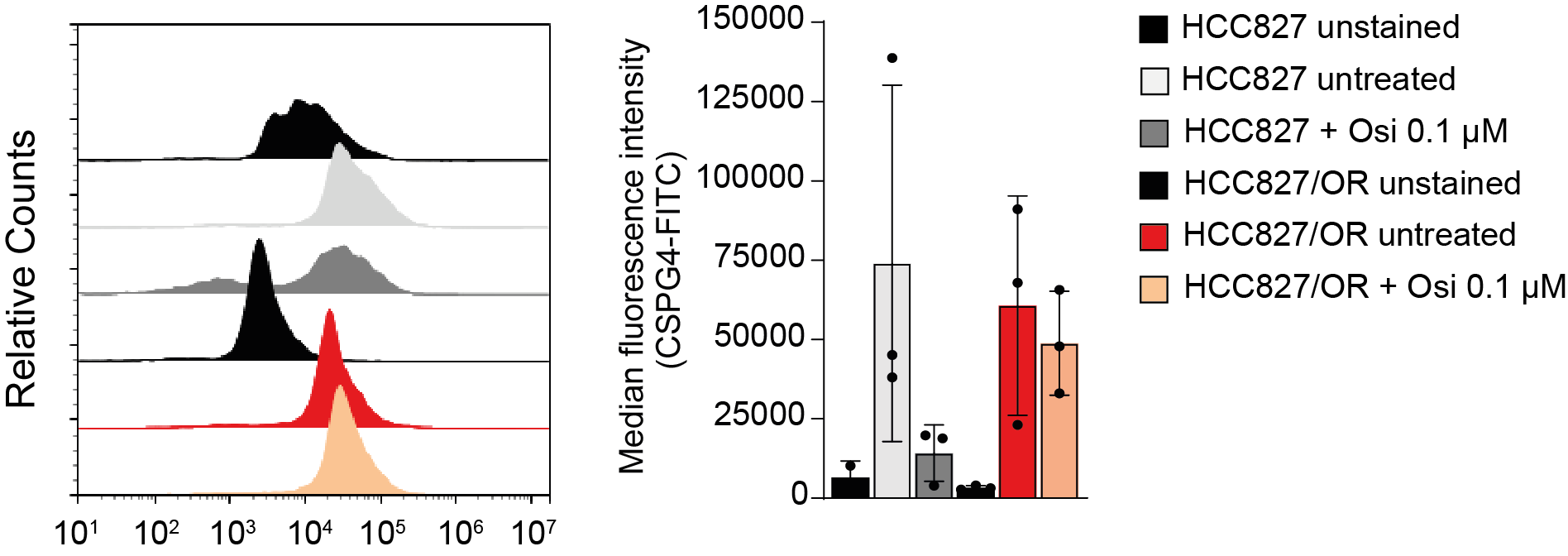
**

**Figure S4 - Chondroitin sulfate proteoglycan expression on mutant *EGFR* expressing non-small cell lung cancer HCC827 cells**. Chondroitin sulfate proteoglycan 4 (CSPG4) cell surface expression was studied in HCC827 or HCC827/OR cells with or without osimertinib treatment (0.1 µM, 48 h), for which non-permeabilized fixed cells were stained by a FITC-conjugated CSPG4 antibody. ***Left panel:*** Histograms of CSPG4-FITC staining from one replicate with the level of autofluorescence of unstained cells is presented as indicated in **Figure 3B**. ***Right panel:*** Quantification of the FITC median fluorescence intensity of CSPG4. Data is based on three experiments and calculated as presented in **Figure 3B**.

**
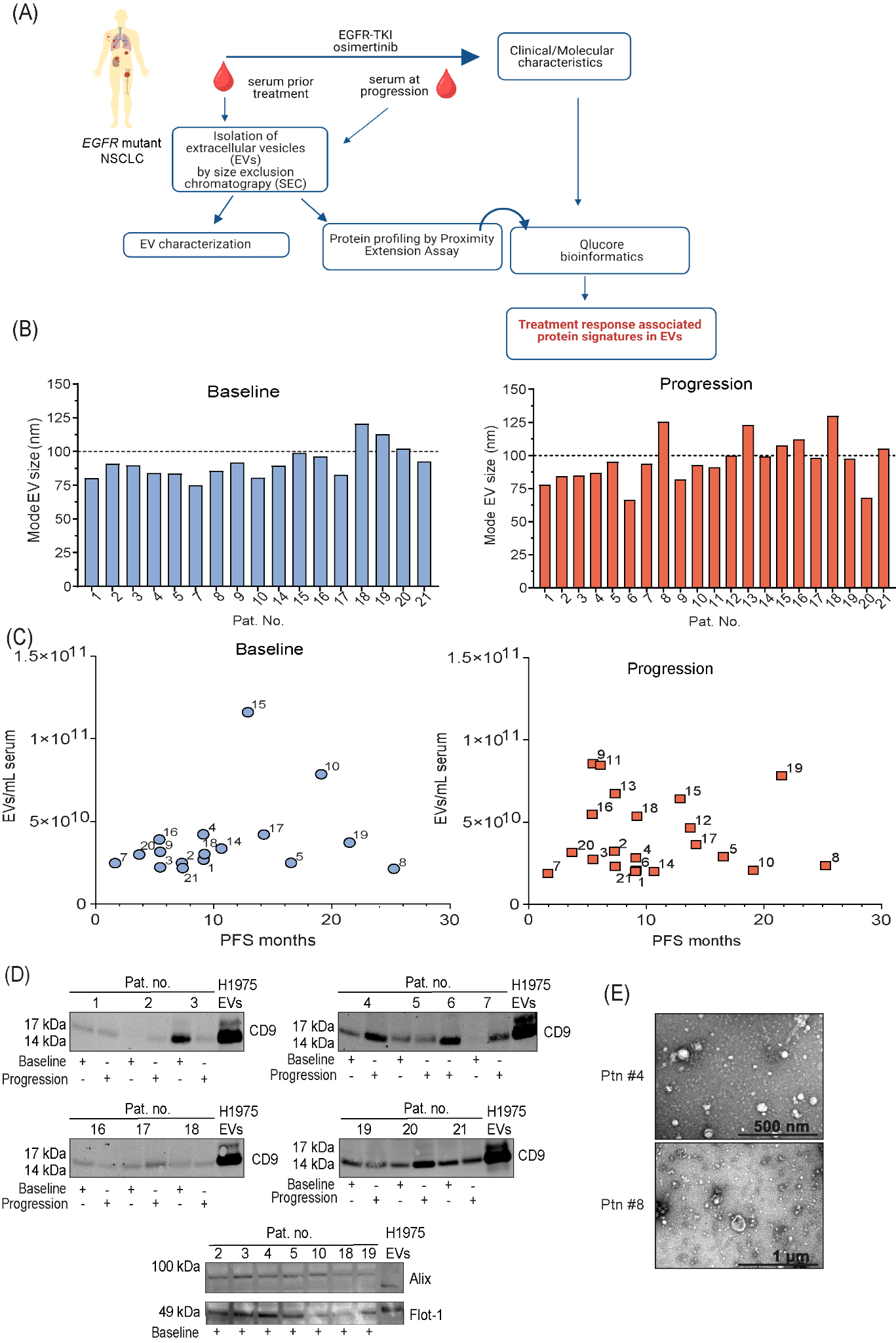
**

**Figure S5 - Characterization of extracellular vesicles obtained from serum of non-small lung cancer patients**. **(A)** Overview of the study. Serum samples were collected at baseline (n=17) or at progression (n=21) on osimertinib from a non-small cell lung cancer patient cohort included in the TREM study, and treated at Karolinska University Hospital (**Table 1**). The extracellular vesicles (EVs) were isolated by size exclusion chromatography (SEC) and the mode particle size (in nm) as well as the number of particles per mL sample analyzed by Nanoparticle tracking analysis (NTA). Expression of CD9, alix and flotillin-1 were confirmed by western blot (WB). EVs were profiled by proximity extension assay (PEA) panels Oncology II^®^ and Immune Oncology^®^. Qlucore^®^ bioinformatics were applied to sort out EV protein cargo profiles. Baseline and progression EV samples of two patients, one with short progression Free Survival (PFS) (Ptn. #3, PFS 5.5 months) and one with long PFS (Ptn. #5, PFS 16.5 months) were analyzed by mass spectrometry. **(B)** The mode size of the EVs of the serum samples of individual TREM patients at baseline (mean value 91.6 (***left panel***)) or progression (mean value 96.2 nm (***right panel***)) was analyzed using NTA. **(C)** EV concentrations in the serum at baseline ranged between 2 x10^10^ to 1 x10^11^ in the baseline samples (***left panel***) and between 2 x10^10^ and 9 x10^10^ EVs/ml at progression. Data was retrieved from the NTA analyses with adjustments made for differences in the serum starting volumes. Data is presented in relation to PFS in months. **(D)** CD9, flotillin-1 (Flot-1) and alix expression in the EVs from the serum samples were analyzed by WB. An equal number of particles as determined by NTA were loaded from each patient sample (4 x10^8^ for CD9 membranes and 1.6 x10^9^ for the alix and Flot-1 membrane respectively). H1975 cell extracts and EVs were used as positive controls. Please note that patient #6 is missing in the baseline cohort as no serum sample was taken. **(E)** The size and morphology of the particles isolated from serum were studied by transition electron microscopy (TEM). Images show particles isolated from serum samples at baseline of two patients Ptn. #4 and #8. The sizes of the particles are indicated by the scale bar.

**
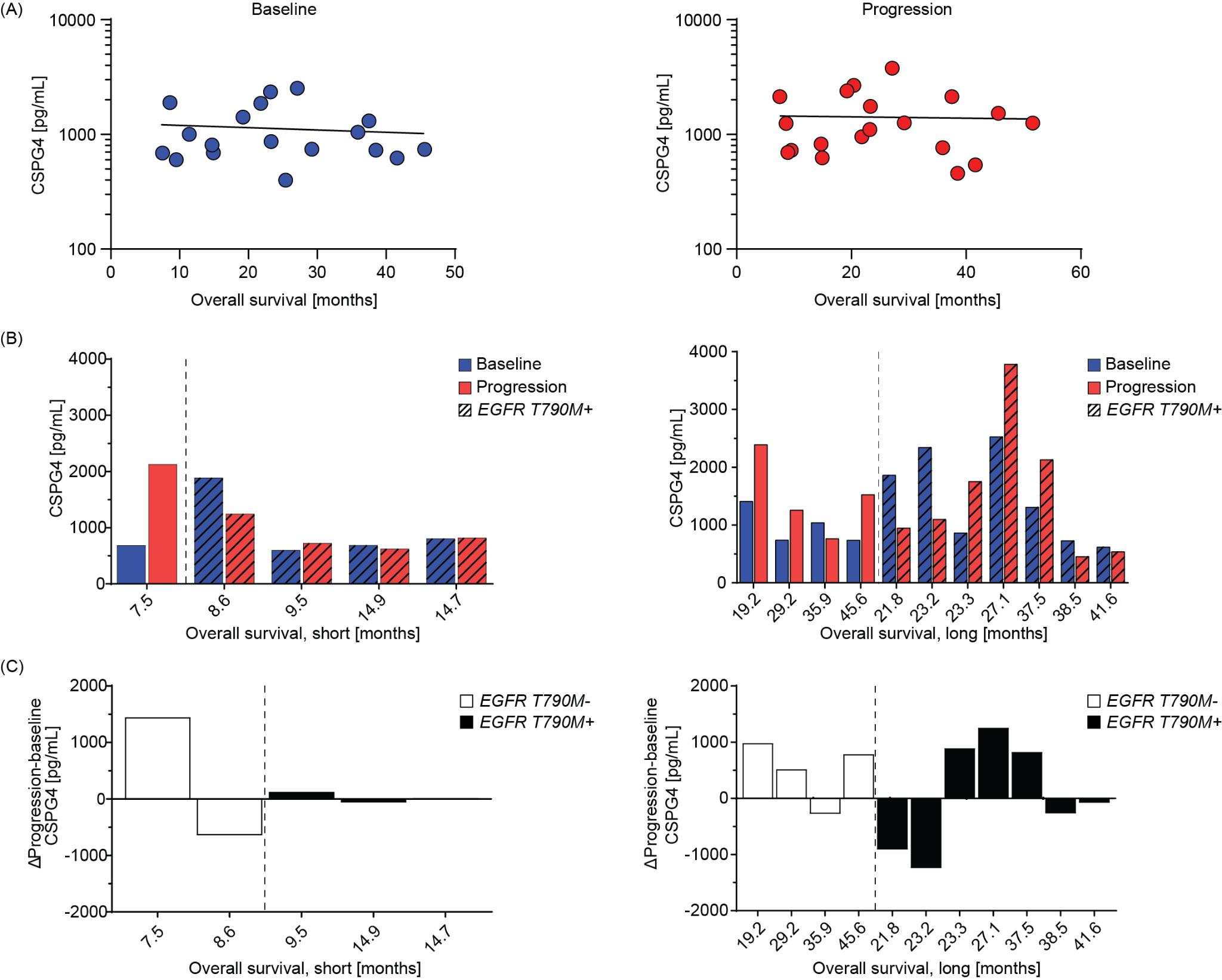
**

**Figure S6 – Expression of chondroitin sulfate proteoglycan 4 in total serum of non-small cell lung cancer patients in relation to overall survival**. Chondroitin sulfate proteoglycan 4 (CSPG4) expression in total serum taken from non-small cell lung cancer (NSCLC) patients at baseline or at progression was studied by enzyme-linked immunosorbent assay (ELISA) (**Table 1**). The CSPG4 concentrations in the samples were all within the standard curve of the assay. **(A)** CSPG4 (pg/mL) in EVs at baseline or at progression for individual patients (monoplicates) are shown with data presented in relation to overall survival (OS) in a linear regression analysis which was not significant. **(B)** Comparison of CSPG4 expression (baseline vs. progression) in serum from NSCLC patients with short OS (less than 17.9 months, ***left panel***) or long OS (above 17.9 months, ***right panel***) (Eide et al. 2020)delimited by the presence or absence of the *EGFR T790M* mutation in the tumor material at study inclusion (no: non-striped bars, yes: striped bars). **(C)** The difference in CSPG4 expression levels in serum at baseline vs*.* progression is presented in relation to OS of the patients and delimited by *EGFR T790M* mutation (no: white bars, yes: black bars).


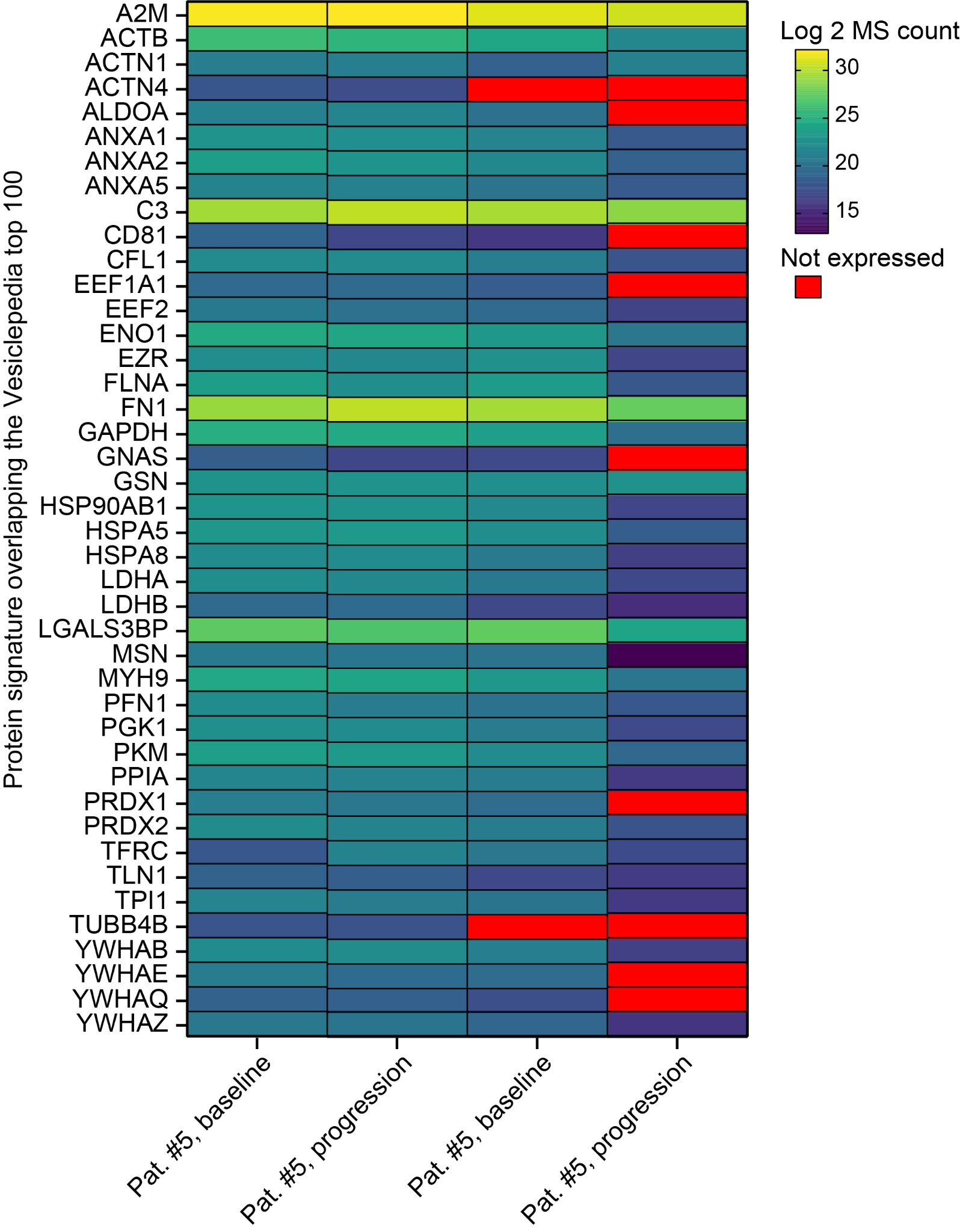


**Figure S7 - Proteins identified in extracellular vesicles from serum of non-small cell lung cancer patients overlapping with Vesiclepedia top 100 list**. Proteins from extracellular vesicles (EVs) isolated from serum samples taken at baseline or progression from Ptn. #3 and Ptn. #5 and which were identified by at least two peptides by mass spectrometry (MS), were searched against the Vesiclepedia top 100 EV protein list (<http://microvesicles.org/extracellular_vesicle_markers>). The proteins in the EVs which overlap with the vesiclepedia proteins are given with their log2 MS count values and presented as a color range of expression. Proteins not detected by MS in individual samples are indicated in red.

**
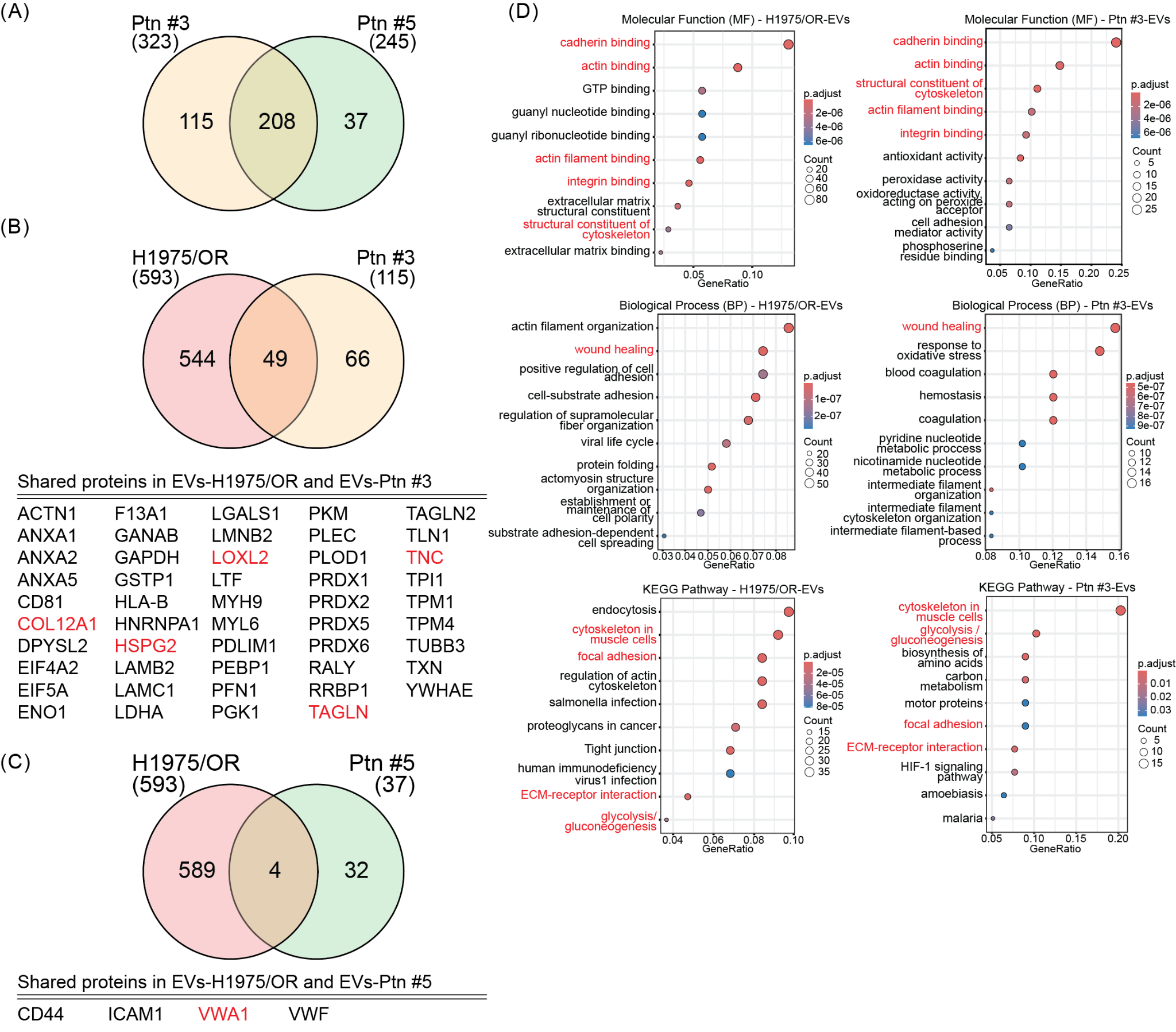
**

**Figure S8 - Mass spectrometry protein profiling of serum-isolated extracellular vesicles protein cargo**. **(A)** Extracellular vesicles (EVs) isolated from serum of two non-small cell lung cancer (NSCLC) patients at baseline, Ptn. #3 (Progression Free Survival (PFS): 5.45 months; Overall Survival (OS): 9.4 months) and Ptn. #5 (PFS: 16.5 months; OS: 37.1 months), were studied by mass spectrometry (MS) for their protein expression and compared in a Venn diagram analysis. **(B-C)** ***Top panel****:* Venn diagram analysis of proteins found to have a higher expression (Log_2_(FC) ≥ 1) in EVs isolated from H1975/OR cell culture media (H1975/OR EVs) as compared to EVs from media of H1975 cells (H1975 EVs) and **(B)** H1975/OR EVs vs. Ptn. #3 EVs and **(C)** vs. Ptn. #5 EVs. ***Bottom panel*:** List of proteins shared between H1975/OR EVs and EVs from serum of Ptn. #3 **(B)** or Ptn. #5 **(C)** at baseline. The EVs proteins associated with osimertinib resistance in H1975/OR (see **Figure 1E-F, Figure 2**) are highlighted in red. **(D)** Enrichment pathway of Gene ontology: molecular function and biological process and KEGG pathways of the samples shown in **(B)** are presented with shared pathways indicated in red.


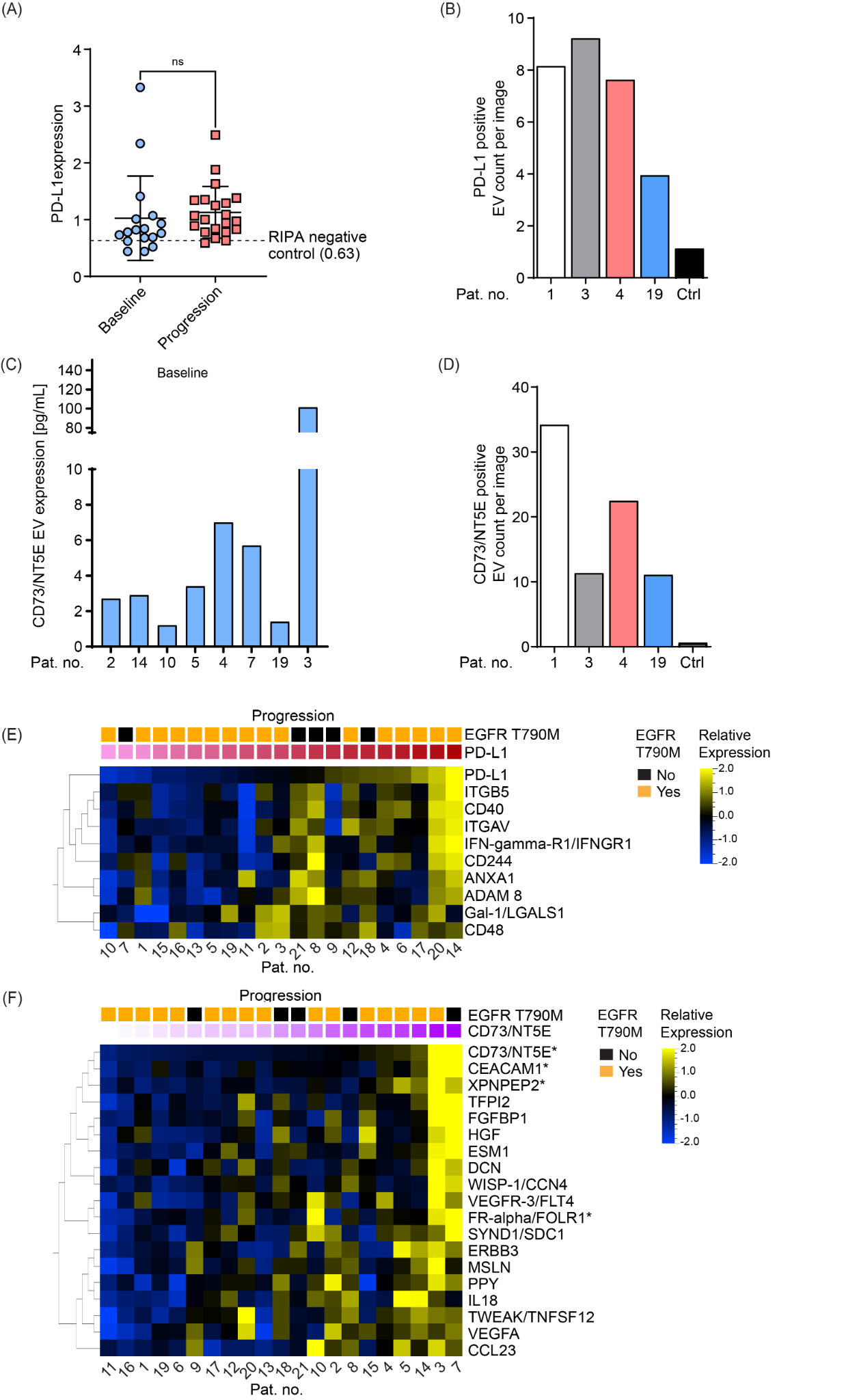


**Figure S9 - Extracellular vesicles isolated from serum of non-small cell lung cancer patients express immune related signaling components**. Extracellular vesicles (EVs) isolated from serum of non-small cell lung cancer patients taken at baseline or progression were analyzed (**Table 1, Figure S6A**). The protein cargo of the EVs were studied by proximity extension assay (PEA) on the Oncology II^®^ and Immuno-oncology^®^ panels with the protein expression values (presented as NPX) further analyzed by the Qlucore^®^ software. Data presented were obtained from EVs isolated from one biological replicate of each serum sample. Note that baseline samples were missing for four patients (Ptn. #6, #11, #12, #13) giving a total of 17 samples. **(A)** The Programmed-death ligand 1 (PD-L1) levels in EVs from the different patient samples were linearized from the NPX values obtained in the PEA assessment. Comparison of levels at baseline or progression revealed no significant difference in expression (Mann-Whitney, two-group analysis). **(B)** PD-L1 expression in EVs from baseline samples were studied by single-EV fluorescence microscopy using a biotinylated-CD9 for capturing the EVs followed by staining with a PE-conjugated PD-L1 antibody. The signals from 40 images (8 images for control sample) were summarized and are presented as the mean number of EVs per image in the individual samples, measured in one technical replica. A sample without EVs served as negative control (Ctrl) for the assay. **(C)** The cluster of differentiation CD73/5'-nucleotidase (CD73/NT5E) expression in individual EVs patient samples isolated from baseline samples was measured by ELISA with values given in pg/mL in 1x10^7^ EVs. **(D)** CD73/NT5E expression on single EVs. EVs from baseline samples of the indicated patients were studied by single-EV fluorescence microscopy analyses using a biotinylated-CD9 for capturing EVs on the cover slip surface followed by staining with an APC-conjugated CD73 antibody. For details of quantification see **(B)** A sample without EVs served as negative control (Ctrl) for the assay. **(E)** A PD-L1 expression-associated protein signature in the serum EVs at progression was obtained by rank regression analysis of the PEA data using the Qlucore^®^ software with a p-value ≤ 0.05, q-value ≤ 0.46. Samples are presented from low PD-L1 (light pink) to high PD-L1 expression (red). **(F)** A CD73/NT5E expression-associated protein signatures in EVs at progression were sorted out by rank regression of the PEA data using Qlucore^®^ software with p-value ≤ 0.01, q-value ≤ 0.06. Samples are presented from low to high CD73/NT5E expression (light purple to dark purple). Proteins which also were present in the EVs at baseline are indicated with an asterix (*).
